# Patched 1 regulates Smoothened by controlling sterol binding to its extracellular cysteine-rich domain

**DOI:** 10.1126/sciadv.abm5563

**Published:** 2022-06-03

**Authors:** Maia Kinnebrew, Rachel E. Woolley, T. Bertie Ansell, Eamon F. X. Byrne, Sara Frigui, Giovanni Luchetti, Ria Sircar, Sigrid Nachtergaele, Laurel Mydock-McGrane, Kathiresan Krishnan, Simon Newstead, Mark S. P. Sansom, Douglas F. Covey, Christian Siebold, Rajat Rohatgi

**Affiliations:** 1 Departments of Biochemistry and Medicine, Stanford University School of Medicine, Stanford, CA, USA.; 2 Division of Structural Biology, Wellcome Centre for Human Genetics, University of Oxford, Oxford, UK.; 3 Department of Biochemistry, University of Oxford, Oxford, UK.; 4 Department of Developmental Biology, Washington School of Medicine, St. Louis, MO, USA.; 5 Kavli Institute for Nanoscience Discovery, University of Oxford, Oxford, UK.; 6 Taylor Family Institute for Innovative Psychiatric Research, Washington University School of Medicine, St. Louis, MO, USA.

## Abstract

Smoothened (SMO) transduces the Hedgehog (Hh) signal across the plasma membrane in response to accessible cholesterol. Cholesterol binds SMO at two sites: one in the extracellular cysteine-rich domain (CRD) and a second in the transmembrane domain (TMD). How these two sterol-binding sites mediate SMO activation in response to the ligand Sonic Hedgehog (SHH) remains unknown. We find that mutations in the CRD (but not the TMD) reduce the fold increase in SMO activity triggered by SHH. SHH also promotes the photocrosslinking of a sterol analog to the CRD in intact cells. In contrast, sterol binding to the TMD site boosts SMO activity regardless of SHH exposure. Mutational and computational analyses show that these sites are in allosteric communication despite being 45 angstroms apart. Hence, sterols function as both SHH-regulated orthosteric ligands at the CRD and allosteric ligands at the TMD to regulate SMO activity and Hh signaling.

## Teaser

Cholesterol activates Hedgehog signaling by binding to the extracellular domain of the Smoothened receptor.

## INTRODUCTION

Signals from the Hedgehog (Hh) pathway are transmitted across the plasma membrane by Smoothened (SMO), a seven-helix transmembrane protein belonging to the G protein–coupled receptor (GPCR) superfamily (*1*). SMO, however, does not directly bind to extracellular ligands of the Hh pathway such as Sonic Hedgehog (SHH). Instead, SHH is received at the cell surface by Patched 1 (PTCH1), a putative cholesterol transporter. In the absence of SHH, PTCH1 inhibits SMO by restricting its access to cholesterol in the membrane of the primary cilium, an antenna-like organelle that projects from the cell surface and functions as a compartment for Hh signaling (*2*, *3*). SHH binds and inhibits PTCH1, thereby raising cholesterol accessibility in the extracellular leaflet of the ciliary membrane (*2*, *4*). Cholesterol can then bind and activate SMO, which transmits the signal to cytoplasmic effectors such as protein kinase A (*5*–*7*). While membrane cholesterol can modulate the activity of many GPCRs (*8*), cholesterol plays a unique role in Hh signaling because it is both necessary and sufficient to activate SMO (*5*). Elucidating the mechanism by which cholesterol activates SMO promises to provide a paradigm for how receptor activity can be controlled by cholesterol.

**Fig. 1.**
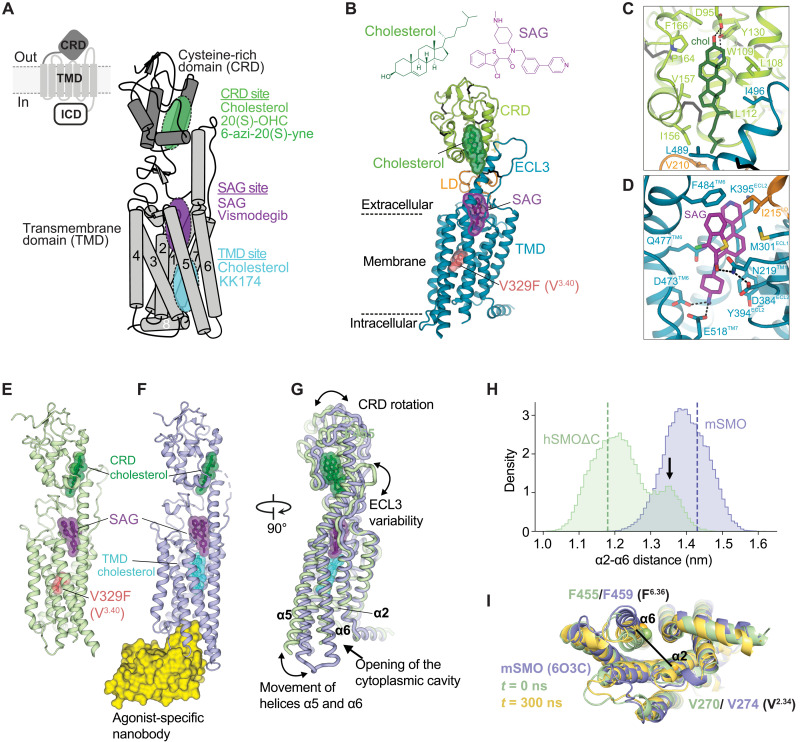
Multiple ligand-binding sites control SMO activation. (**A**) SMO is composed of a CRD, TMD, and intracellular domain (ICD). Schematic of mSMO highlighting three ligand-binding sites (the CRD, SAG, and TMD sites) along with interacting ligands. (**B**) Structure of hSMOΔC-BRIL-V329F in complex with cholesterol and SAG, with close-ups shown in (**C**) and (**D**). (**E** to **G**) Superposition (G) of the hSMOΔC-BRIL-V329F:cholesterol:SAG complex (E) with the complex of mSMO with SAG, cholesterol, and a nanobody (Nb8) (PDB 6O3C, *13*) (F). (F) is considered an active-state SMO structure. (**H**) Histogram of the distances between the Cα atoms of hSMO V270 (V^2.34^) on helix α2 and hSMO F455 (F^6.36^) on helix α6 in atomistic simulations of mSMO (blue) and hSMOΔC (green), each bound to SAG and CRD cholesterol. Dashed lines indicate the starting α2 to α6 distances, and the arrow indicates the increased distance between α2 and α6 caused by outward movement of α6 (fig. S1). (**I**) Snapshots showing the outward movement of α6 in hSMOΔC (yellow, fig. S1D). The structure in green shows the distance between α2 and α6 at the start of the hSMOΔC simulation, and the structure in blue shows active-state mSMO [Protein Data Bank (PDB) 6O3C] (fig. S1).

While SMO is clearly a sterol-responsive protein (*9*), its mechanism of activation is a topic of debate. In solving a crystal structure of SMO that included both its transmembrane domain (TMD) and extracellular cysteine-rich domain (CRD), we unexpectedly found a cholesterol molecule bound in a shallow hydrophobic groove in the CRD, positioned >10 Å above the extracellular leaflet of the plasma membrane (Fig. 1A) (*7*, *10*). This same groove is used by Frizzleds, the closest relatives to SMO in the class F GPCRs, to bind to the fatty acid attached to WNT ligands (*11*). Ligand affinity assays confirmed that cholesterol can bind to the SMO CRD in solution and structure-guided mutations that abrogated binding impaired signaling by SHH in both cultured cells and mouse embryos (*5*, *6*, *12*). Together, these results led us to suggest that SHH, by inactivating PTCH1, promotes cholesterol binding to the SMO CRD.

To stabilize SMO for structural studies, we included an inactivating mutation in the TMD (*7*). This mutation changed valine 329 (V^3.40^ in the class F Ballesteros-Weinstein nomenclature) in human SMO (hSMO) to phenylalanine (V329F), which corresponds to the V333F mutation in mouse SMO (mSMO). A subsequent crystal structure of mSMO lacking this mutation in complex with the synthetic agonist SAG bound at the extracellular end of the TMD and the Nb8 nanobody (that recognizes the active conformation of SMO) bound to the intracellular surface confirmed the presence of the CRD-bound cholesterol (*13*). This structure also revealed a second cholesterol molecule bound in the TMD positioned just below SAG (Fig. 1A). While sterol binding to the TMD has not been demonstrated in solution, mutations (such as V333F) predicted to prevent sterol binding to the TMD abolish SMO activity in cultured cells (*7*, *13*, *14*). On the basis of cryo–electron microscopy structures, a related model suggested that cholesterol molecules occupy various positions in a putative tunnel that extends from the TMD site to the CRD site (*15*). Simulation studies suggest that cholesterol molecules may also bind to the lipid-facing surface of the TMD, in a crevice between helices α2 and α3 adjacent to the TMD site (*16*).

The structural and mutational data summarized above raise the following questions: Why does SMO have two (or more) cholesterol-binding sites? While cholesterol is likely to be the physiological ligand at both sites, which of these sites is the orthosteric site (defined as the signal-controlled site in SMO that is regulated by PTCH1)? Comparisons between prior studies arguing for the importance of one site over the other are hampered by differences in the mutations introduced at the two sites, differences in the expression systems used for SMO mutants, and differences in the assays used to measure signaling strength (*5*–*7*, *13*, *17*). For example, studies of the CRD site used stable expression of mSMO mutants and measured the transcription of the endogenous Hh target gene *Gli1*. In comparison, analysis of the TMD site used transient overexpression of mSMO mutants in conjunction with a synthetic luciferase-based reporter system. To understand the roles of the CRD and TMD sites in SMO activation and its regulation by PTCH1, we took three different approaches that rely on structure-guided mutagenesis and new sterol analogs that selectively engage the TMD and CRD sites. Our results support the model that sterol occupancy of both sites is required for maximum SMO activity. The TMD site functions as an allosteric site that influences the magnitude of both basal and SHH-induced SMO activity, while the CRD site is the primary orthosteric site that controls the SHH-driven increase in SMO activity. Thus, cholesterol activates SMO by binding to two physically distant sites on a GPCR family protein that communicate with each other through an allosteric linkage.

## RESULTS

### Multiple ligand-binding sites can regulate SMO activity

Our experiments focused on the SMO ligand–binding sites depicted in Fig. 1A: the two sterol-binding sites, referred to as the CRD and the TMD sites, and the SAG-binding site that sits just above the TMD site closer to the extracellular side of the membrane (*10*, *18*, *19*). The V333F mutation (V329F in human numbering or V^3.40^F) in the TMD site abolishes mSMO activation by the native ligand SHH, an observation used to argue that the TMD site is more important for SMO regulation by PTCH1 (*13*). However, V333F may simply stabilize mSMO in an inactive state. Consistent with this possibility, mSMO-V333F cannot be activated by any SMO agonists, including those that directly bind to the SAG or the CRD sites (*7*). mSMO-V333F fails to localize in primary cilia in response to SHH, a compartment where SMO must accumulate to activate downstream Hh signaling (fig. S1, A and B).

To understand the relationship between ligand binding and activation, we determined the crystal structure of hSMOΔC-BRIL-V329F in a complex with cholesterol (bound to the CRD site) and SAG at 3.0-Å resolution (Fig. 1B, table S1). This hSMO protein [identical to the one used in our prior structural work (*7*)] lacks the disordered C-terminal intracellular domain (Fig. 1A) and carries both the inactivating V329F mutation and a BRIL domain inserted in the third intracellular loop to facilitate crystallization. Cholesterol and SAG occupy positions in our structure similar to those reported for complexes of SMO with SAG alone or cholesterol alone (*7*, *19*), showing that these two agonists can bind even when SMO is mutationally stabilized in an inactive state (Fig. 1, C and D). We compared our inactive-state structure to the structure of mSMO bound to SAG, cholesterol, and a nanobody (Nb8) raised against activated (SAG-bound) SMO (Fig. 1, E to G) (*13*). The latter structure was proposed to represent an active-state structure because it revealed an outward movement of the intracellular ends of the helices α5 and α6, considered to be a hallmark of GPCR activation that allows coupling to downstream signaling components in the cytoplasm. Opening of the cytoplasmic face of the TMD, stabilized by Nb8 binding, was associated with binding of a second cholesterol molecule to the TMD site (Fig. 1F). Consistent with functional data showing that the V333F mutation locks mSMO in an inactive state regardless of ligand binding (fig. S1, A and B), the α5 and α6 helices in our structure were positioned in a more inward orientation closer to the central axis of the TMD (Fig. 1G).

With these high-resolution structures as starting points, we performed atomistic simulations of the hSMO and mSMO structures. We reverted the phenylalanine mutation at residue 329 in hSMO to the wild-type (WT) valine and removed Nb8 from mSMO during these simulations to probe the effects of ligand binding on the α5- and α6-helical movements associated with GPCR activation. The outward movement of α5/α6 in the mSMO:SAG:cholesterol:Nb8 structure causes an increased distance between helices α2 and α5/α6, hereafter referred to as the “open” active conformation (Fig. 1H). mSMO remained in the open conformation, regardless of whether it was bound to SAG and CRD-cholesterol, CRD-cholesterol only, or present in the ligand-free *apo* state (Fig. 1H and fig. S1, E and F). In contrast, one replicate simulation of hSMO bound to SAG and CRD-cholesterol (that started with SMO in a closed conformation) showed partial activation as measured by outward movement of the α5 and α6 helices (Fig. 1, H and I, and fig. S1, C and D). While it is rare to observe substantial conformational changes at atomistic simulation time scales (in the absence of enhanced sampling), these intermediary conformations have been previously observed in equilibrium simulations of other GPCR systems (*20*, *21*). Together, these structural and computational analyses suggested that SMO can adopt an active-state conformation even without cholesterol bound to the TMD site, which has been presented as an obligate driver of SMO activation (*13*, *15*). Thus, we decided to experimentally test the requirement of the CRD and TMD cholesterol–binding sites in SMO activation in response to SHH using diverse approaches.

### The CRD site mediates SHH-induced increases in SMO activity

**Fig. 2.**
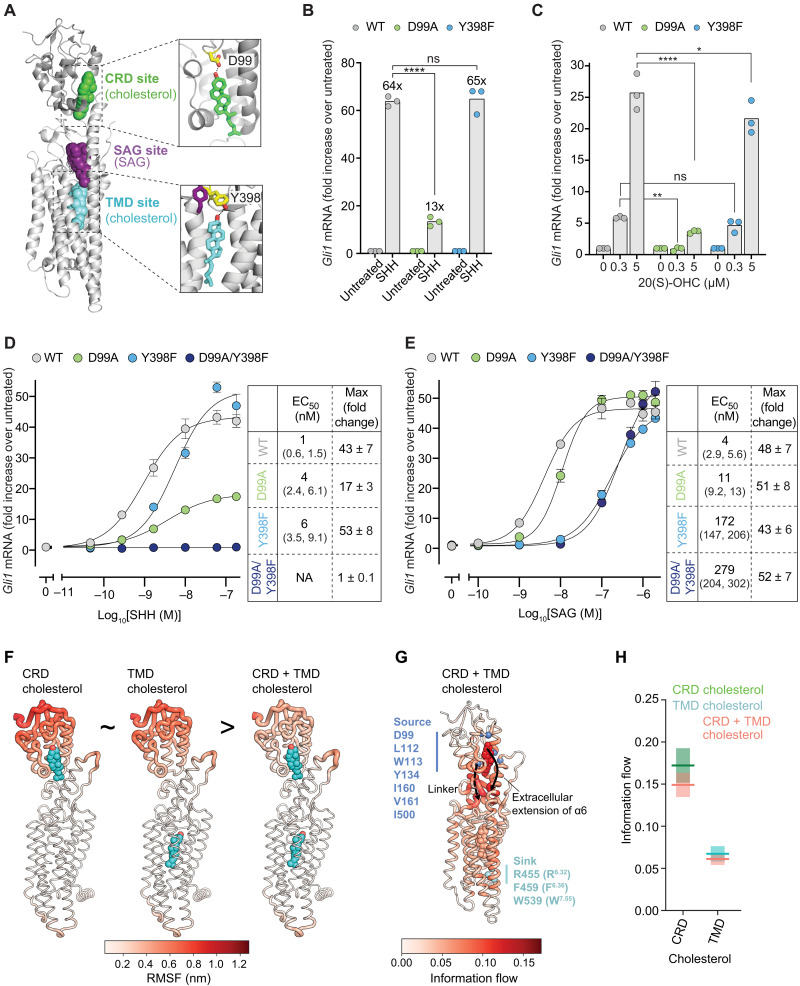
The CRD mediates the fold increase in SMO activity triggered by SHH. (**A**) Close-ups of cholesterol bound to the CRD and TMD sites of mSMO (PDB 6O3C). D99 in the CRD and Y398 in the TMD make hydrogen bonds with the 3β-hydroxyl of cholesterol. (**B** and **C**) Fold change in endogenous *Gli1* mRNA abundance in response to SHH (50 nM for 20 hours) (B) or 20(S)-OHC (20 hours) (C) in *Smo*^−/−^ cells stably expressing the indicated mSMO variants. ns, not significant. (**D** and **E**) Dose response curves for SHH (D) and SAG (E) in *Smo*^−/−^ cells stably expressing the indicated mSMO variants (20 hour treatment). Tables list the EC_50_ (with 95% confidence intervals) and the maximum fold change (with SEM). NA, not applicable. (**F**) RMSF in residues of mSMO bound to the indicated ligands during simulations are mapped onto the mSMO structure and colored from low (white) to high (red) fluctuation. (**G**) Information flow from source residues lining the CRD pocket (blue) to sink residues on α6/7 (cyan) is colored from regions of low (white) to high (red) information flow. Cholesterol molecules are also colored on the basis of information flow. (**H**) Information flow through the cholesterol molecules in simulations of mSMO initiated with cholesterol bound to the CRD alone, the TMD alone, or both. Exact *P* values for comparisons: (B) WT versus D99A (+SHH) < 0.0001 and WT versus Y398F (+SHH) = 0.7545. (C) WT versus D99A [0.3 mM 20(S)-OHC] = 0.0057, WT versus D99A [5 mM 20(S)-OHC] < 0.0001, WT versus Y398F [0.3 mM 20(S)-OHC] = 0.7891, and WT versus Y398F [5 uM 20(S)-OHC] = 0.0399. Experiments shown in (B) to (E) were performed three different times with similar results.

Which of the cholesterol-binding sites are regulated by PTCH1? In other words, which site becomes occupied by cholesterol when SHH binds and inactivates PTCH1? To address this question, we generated structure-guided mutations in the CRD and TMD sites of mSMO designed to disrupt hydrogen-bonding interactions with the 3β-hydroxyl of cholesterol: D99A in the CRD site and Y398F in the TMD site (Fig. 2A). The D99A mutation in the mSMO CRD (D95A in hSMO) abrogated binding of purified hSMOΔC-BRIL-V329F to cholesterol in a previously validated ligand affinity assay (fig. S2B) (*7*). While the field lacks an analogous solution–based assay to measure the interaction between cholesterol and the TMD site in SMO, atomistic simulations confirmed that Y398 in mSMO engages in a hydrogen-bonding interaction with the 3β-hydroxyl of cholesterol (Fig. 2A and fig. S2C).

These mutants were stably expressed in *Smo*^−/−^ cells to exclude signaling from endogenous WT mSMO. The abundances of these mutant proteins in the whole cell and the primary cilium were comparable (fig. S2, D to F). To evaluate the change in the signaling activity of mSMO variants in response to SHH, we measured the fold increase in the abundance of endogenous *Gli1* mRNA, a direct transcriptional target of Hh signaling. The fold change in *Gli1* mRNA abundance (rather than the absolute magnitude of *Gli1* mRNA abundance) is the best metric for SHH-induced changes in SMO activity since it will be less sensitive to changes in SMO protein abundance, the SMO conformational ensemble, or SMO subcellular localization (all of which can be altered as unintended consequences of mutations). For example, if a mutation reduces both basal and SHH-stimulated SMO signaling by the same magnitude, then the fold increase in SMO signaling (the ratio of SHH-stimulated to basal activity) will remain unchanged. Such a mutation may affect SMO abundance and SMO subcellular localization or bias the SMO conformational ensemble toward inactive states but probably does not affect the SHH-driven change in SMO activity.

The D99A mutation in the CRD site impaired SHH-induced mSMO activation, reducing *Gli1* induction from 64- to 13-fold (Fig. 2B). In contrast, mSMO-Y398F, which carries a corresponding mutation in the TMD site, remained fully responsive to SHH, suggesting that this site is less important for the fold increase in mSMO activity seen when PTCH1 is inactivated by SHH (Fig. 2B). mSMO-WT and mSMO-Y398F were also equally responsive to 20(S)-hydroxycholesterol [20(S)-OHC], a CRD ligand whose activity is reduced by the D99A mutation (Fig. 2C) (*5*, *7*). In summary, the Y398F mutation in the TMD site did not change the fold increase in *Gli1* abundance triggered by either CRD ligands or inactivation of PTCH1 by SHH.

To further understand the relationship between the CRD and TMD cholesterol–binding sites, we measured the response of mSMO-D99A and mSMO-Y398F to increasing concentrations of SHH and SAG (Fig. 2, D and E). The Y398F TMD mutation reduced the potency of SHH, measured by the concentration of SHH required to half-maximally increase *Gli1* mRNA [also called the median effective concentration (EC_50_)], but did not change the maximum fold increase in *Gli1* abundance (known as the efficacy) (Fig. 2D). In contrast, both the potency and the efficacy of SHH were reduced by the D99A CRD mutation. The residual SHH responsiveness seen in mSMO-D99A could be caused by two reasons: (i) This mutation fails to completely eliminate cholesterol binding to the CRD in cells, or (ii) SHH (acting indirectly through PTCH1) also regulates cholesterol accessibility to the TMD site. Introduction of a second mutation (Y134F) in the hydrogen-bonding network that positions cholesterol in the CRD (fig. S2A) further reduced responsiveness to both SHH and 20(S)-OHC, highlighting the central regulatory role of the mSMO CRD in PTCH1 regulation (fig. S2, G and H).

To test potential long-range interactions between the CRD and TMD sites, located 45 Å apart, we compared the signaling activity of the D99A and Y398F single mutants to the mSMO-D99A/Y398F double mutant (formally known as a double-mutant cycle analysis). mSMO-D99A/Y398F was completely unresponsive to SHH (Fig. 2D). In a key control that demonstrated protein integrity, all three mutant mSMO proteins supported the same maximum fold increase in *Gli1* mRNA in response to SAG (Fig. 2E). The reduced potency of SAG in cells expressing mSMO-Y398F and mSMO-D99A/Y398F is likely related to the proximity of the Y398 residue to the SAG-binding site (Fig. 2A). Given that we lack an assay to measure binding of cholesterol to the TMD, the marked lack of SHH-induced signaling activity in mSMO-D99A/Y398F provides indirect (but important) evidence that the Y398F mutation disrupts the TMD site.

If the CRD and TMD-binding sites were independent, then we would predict an additive decrease in SHH responsiveness in the mSMO-D99A/Y398F double mutant compared to the individual single mutants. Purely additive effects of the D99A and Y398F mutations would predict an EC_50_ of ~24 nM and a maximum induction of ~20-fold in the D99A/Y398F double mutant. However, the complete abrogation of activity in the double mutant represents a greater than additive effect and provides evidence for long-range allosteric communication between the CRD and TMD sites. This allosteric interaction explains prior observations that mutations in the TMD site can reduce the potency of SHH even if SHH regulates cholesterol occupancy of the CRD (Fig. 2D) (*13*).

Last, we performed atomistic simulations of mSMO in the ligand-free state or with cholesterol bound to the CRD site alone, the TMD site alone or both sites simultaneously. To compare the dynamic movements of mSMO domains in these four states, we calculated the root mean square fluctuation (RMSF) of residues across the simulations. RMSF values for the CRD were similar when a single cholesterol was bound to either the CRD or TMD (Fig. 2F). In contrast, when both sites were occupied, the CRD fluctuations were markedly reduced, suggesting cooperative interactions between the CRD and TMD sites. To further dissect this observation, we calculated the flow of information (also known as current) through mSMO using a recently developed method for calculation of allostery that can include lipids in pathway calculations (*22*). Two main pathways of information flow between the CRD pocket and α6/α7 were revealed: (i) the extracellular extension of α6 (Fig. 2G, solid arrow) and (ii) a ladder of residues on the opposing CRD face and the mSMO linker domain (Fig. 2G, dashed arrow). The CRD-bound cholesterol is a major contributor to the information flow, with one of the highest flow values compared to all other residue and lipid nodes (Fig. 2, G and H). The contribution of the TMD cholesterol to the information flow is lower than that of the CRD cholesterol. When both the TMD and CRD cholesterol sites are occupied, information flow through the CRD cholesterol is reduced, while the TMD cholesterol is unaffected, consistent with the role of the TMD cholesterol in modulating signaling activity that propagates from the CRD cholesterol (Fig. 2H). Cholesterol binding to the CRD reduces information flow through residues in the CRD face/linker region (fig. S3, A and B). In contrast, information flow through a subset of residues on the α6 extension or lining the helical bundle (including Y398) is enhanced upon CRD cholesterol binding (fig. S3, A and B). This suggests that cholesterol binding to SMO favors information flow through the α6/TMD pathway, while the CRD face/linker pathway is more important in the *apo* state. An additional intermediate sterol-binding site has been proposed in proximity to the linker region of SMO (*15*). However, we did not find cholesterol binding to this site to be stable in simulations. The presence of this third cholesterol destabilized both the CRD and the TMD-bound cholesterol molecules in SMO (fig. S3, C to H).

Together, this analysis suggests that the CRD cholesterol–binding site is primarily responsible for the increase in SMO activity when PTCH1 is inactivated by SHH. The TMD functions as a long-range allosteric site to tune SMO activity.

### The TMD site regulates basal SMO signaling in the absence of SHH

SMO, similar to many GPCRs, demonstrates basal signaling activity even in the absence of activating ligands. Ligand-independent signaling activity is explained by the fact that GPCRs are not on-off switches but rather populate an ensemble of conformations determined by a free energy landscape (*23*). Receptor conformations of lower energy are more populated; ligands (including lipids) can alter this free energy landscape to favor a population of conformations with specific signaling activities (*24*, *25*). Basal receptor activity is proposed to be a consequence of the fact that even in the absence of agonists, a small number of receptors at any given snapshot in time are in an active conformation.

**Fig. 3.**
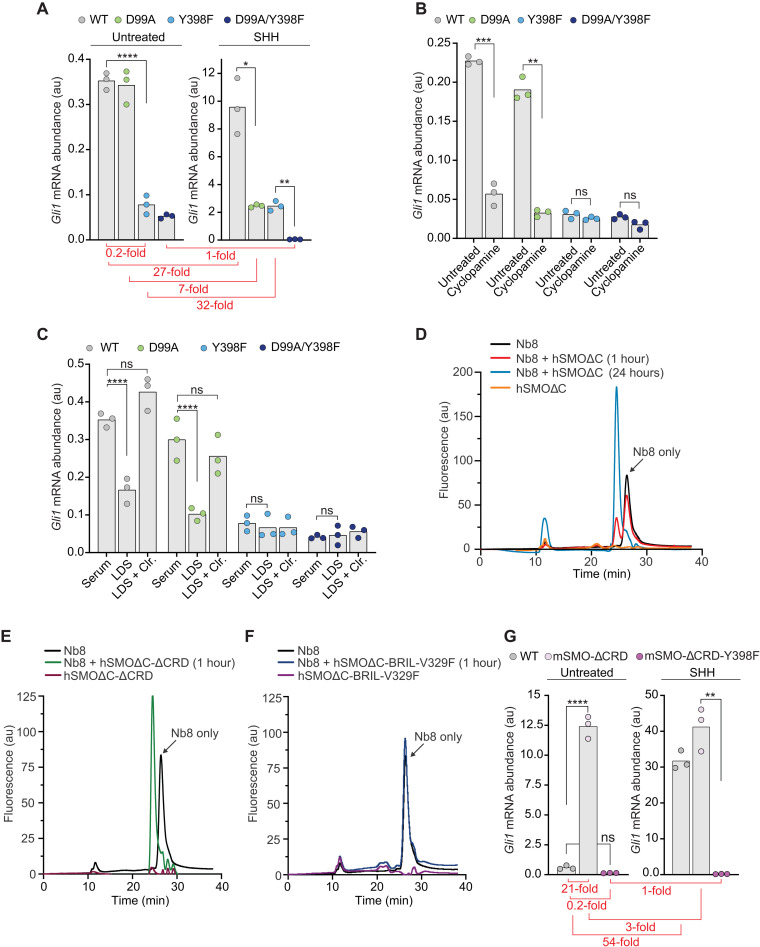
The TMD site regulates basal SMO activity in the absence of SHH. (**A** and **B**) Absolute *Gli1* mRNA abundance (au, arbitrary units) in the presence and absence of SHH (50 nM) (A) or cyclopamine (5 μM) (B) in *Smo*^−/−^ cells stably expressing the indicated mSMO variants. (**C**) *Gli1* mRNA abundances in the absence of SHH (a measure of basal SMO activity) in medium supplemented with fetal bovine serum (Serum), lipoprotein-depleted fetal bovine serum (LDS), or LDS supplemented with 10 μM MβCD:cholesterol (Clr.). (**D** to **F**) Fluorescence-detection size exclusion chromatography (FSEC) was used to assess binding of Nb8-mVenus (*13*) to the indicated hSMO variants after incubation for 1 hour or 24 hours. Binding to hSMO causes a shift in the Nb8-mVenus elution profile to a higher apparent molecular weight (earlier elution time). (**G**) *Gli1* mRNA abundance in the presence and absence of SHH (50 nM) in *Smo*^−/−^ cells stably expressing mSMO-WT, mSMO-ΔCRD, or mSMO-ΔCRD-Y398F. The duration of drug treatment in (A), (B), (C), and (G) was 20 hours. The *y* axes of the graphs without (left) and with (right) SHH in (A) and (G) are different to clearly show basal mSMO signaling activity. The fold change (SHH-stimulated *Gli1* divided by basal *Gli1*) for each of the mSMO variants is depicted in red in (A) and (G). Exact *P* values for comparisons: (A) WT versus Y398F (untreated) < 0.0001, WT versus D99A (+SHH) = 0.0255, and Y398F versus D99A/Y398F (+SHH) = 0.0068. (B) WT untreated versus cyclopamine = 0.0009, D99A untreated versus cyclopamine = 0.0013, Y398F untreated versus cyclopamine = 0.2262, and D99A/Y398F untreated versus cyclopamine = 0.0622. (C) WT serum versus LDS < 0.0001, WT serum versus LDS + cholesterol = 0.0604, D99A serum versus LDS < 0.0001, D99A serum versus LDS + cholesterol = 0.4310, Y398F serum versus LDS = 0.6333, and D99A/Y398F serum versus LDS = 0.7885. (G) WT versus mSMO-ΔCRD (untreated) < 0.0001, WT versus mSMO-ΔCRD-Y398F (untreated) = 0.5700, and mSMO-ΔCRD versus mSMO-ΔCRD-Y398F (+SHH) = 0.0073.

The absolute abundance of *Gli1* in the absence of SHH can be used as a measure of basal SMO activity. While the basal signaling activities of mSMO-WT and mSMO-D99A were comparable, mSMO-Y398F and mSMO-D99A/Y398F were both five times less active in the absence of SHH (Fig. 3A). Since the maximum SHH-stimulated *Gli1* abundance was also reduced by a similar magnitude, the fold increase in mSMO-Y398F activity remained unchanged. This analysis shows that only measuring the absolute level of a transcriptional reporter such as *Gli1*, without accounting for differences in basal reporter activity, will lead to the erroneous conclusion that the Y398F mutation in mSMO compromises SHH responsiveness. The Y398F mutation likely alters the energy landscape of mSMO such that both basal and stimulated signaling activities are reduced, but the change in signaling activity induced by SHH is unaltered.

Cyclopamine, a sterol-like inhibitor of mSMO, is an inverse agonist that suppresses the basal activity of mSMO-WT and mSMO-D99A (Fig. 3B) (*26*). However, cyclopamine had no effect on the basal activity of mSMO-Y398F (Fig. 3B). In an important control, cyclopamine could still inhibit mSMO-Y398F signaling in response to SHH, showing that this mutation did not simply prevent the interaction between SMO and cyclopamine (fig. S4A). These data suggested that constitutive cholesterol occupancy of the TMD site sets the basal level of SMO signaling. In support of this idea, depletion of membrane cholesterol reduced the basal activities of mSMO-WT and mSMO-D99A to the level seen in mSMO-Y398F (Fig. 3C). Cholesterol depletion had no effect on the basal activity of mSMO-Y398F.

A truncation mutant of mSMO lacking the entire CRD (mSMO-ΔCRD) displays much higher basal signaling compared to mSMO-WT when expressed in cells (*7*). We previously proposed that CRD interactions with the TMD and the linker domain stabilize the inactive state of SMO (*7*). To directly test the role of the CRD in controlling SMO activity, we fused the active-state selective Nb8 to mVenus (Nb8-mVenus) (*13*) and probed its interactions with nonfluorescent variants of hSMOΔC using fluorescence-detection size exclusion chromatography (FSEC). Nb8 was initially selected to preferentially bind an activated conformation of mSMO, one in which the α5/α6 helices are displaced outward (Fig. 1F). The stable interaction of Nb8-mVenus with hSMOΔC will induce a shift to higher molecular weights (shorter retention times) on the FSEC trace. In the absence of any added ligands, only a fraction of hSMOΔC formed a complex with Nb8 after a 1-hour incubation, as shown by a double peak in the FSEC trace (Fig. 3D). However, a longer incubation of 24 hours allowed the entire population of hSMOΔC to bind Nb8-mVenus. The slow kinetics of binding likely reflect the slow spontaneous conversion of hSMOΔC to an active conformation, which is recognized and then stabilized by Nb8 binding. The fraction of hSMOΔC that is bound to Nb8 at 1 hour likely represents molecules that adopted an activated conformation during purification. In contrast, the entire population of Nb8-mVenus shifted to a higher molecular weight within 1 hour when incubated with hSMOΔC lacking the CRD (hSMOΔC-ΔCRD), demonstrating much more rapid binding of hSMOΔC to the active-state Nb8 when the CRD is removed (Fig. 3E). In a control experiment, we did not detect an interaction between Nb8-mVenus and the inactive hSMOΔC-BRIL-V329F (Fig. 3F) (*7*). Using a completely purified system, these data show a direct role of the CRD in preventing the TMD from adopting an active state (and consequently triggering downstream cytoplasmic signal propagation).

Given this key role of the CRD in suppressing TMD activity, mSMO-ΔCRD provided an opportunity to study the TMD site in isolation. Similar to its effect on mSMO-WT (Fig. 3A), the Y398F mutation markedly reduced the basal activity of mSMO-ΔCRD (Fig. 3G). As reported previously, mSMO-ΔCRD signaling is increased modestly by SHH (an observation that has been used to argue that the TMD site is the major PTCH1-regulated site) (Fig. 3G) (*13*, *27*). The Y398F mutation also eliminated SHH responsiveness of mSMO-ΔCRD. Thus, the integrity of the TMD site is required for both the high basal activity and the SHH responsiveness of mSMO-ΔCRD. These results also confirm that the Y398F mutation (which has no effect on the SHH-induced fold increase in mSMO activity; Fig. 2B) disrupts the sterol-binding site in the TMD. In important controls, the abundance of mSMO-ΔCRD-Y398F was similar to mSMO-ΔCRD in our cell lines (fig. S4B), and mSMO-ΔCRD-Y398F could be activated by SAG (showing protein integrity) but not by cholesterol (fig. S4, C and D).

Our results suggest that constitutive cholesterol binding to the TMD site in SMO changes the energy landscape of SMO such that basal signaling activity is increased. The CRD provides a restraining influence, preventing full SMO activation, until SHH (by inactivating PTCH1) increases outer leaflet cholesterol accessibility to the point that the CRD site is occupied (*4*). This model explains the modest SHH responsiveness of mSMO-ΔCRD: An increase in cholesterol accessibility triggered by SHH will lead to greater occupancy of the TMD site and hence an increase in signaling activity.

### The CRD site is required for SHH-induced SMO activation

Our previous experiments showed that the increase in SMO activity in response to SHH depends critically on the CRD cholesterol–binding site. However, mSMO carrying mutations in the CRD site retains some SHH responsiveness (Fig. 2D), as has been noted previously (*17*). Perhaps cholesterol occupancy of both the CRD and TMD sites is regulated by PTCH1?

**Fig. 4.**
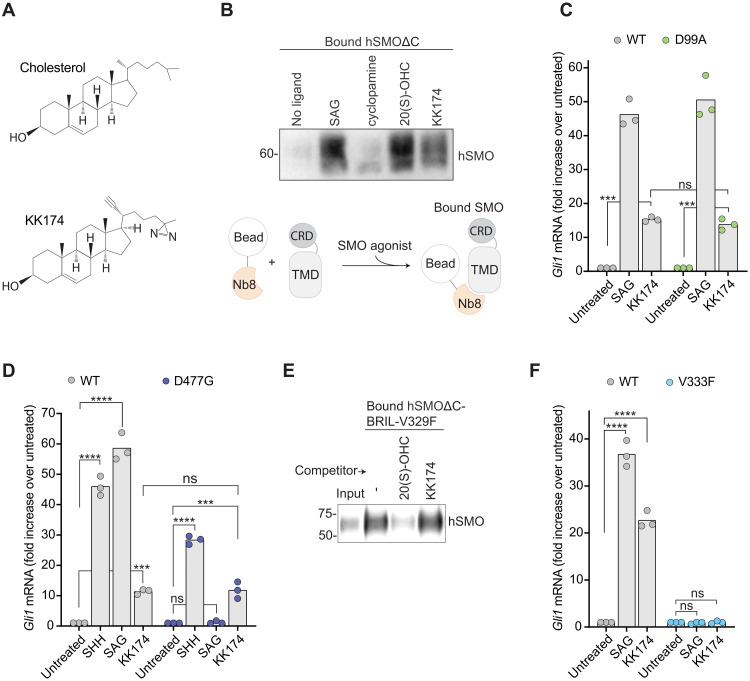
KK174 is a SMO agonist that functions at the TMD. (**A**) Structures of cholesterol and KK174. (**B**) The interaction between hSMOΔC and Nb8 was assessed using a pull-down assay in the presence of the indicated SMO ligands (100 μM each for 16 hours). Immunoblot shows the amount of hSMOΔC that coprecipitates with Nb8 captured on beads. Figure S5A shows the abundance of SMO in flow-through samples from this pull-down. (**C**, **D**, and **F**) Fold increase in *Gli1* mRNA induced by the addition of the indicated SMO agonists (100 nM SAG, 300 μM MβCD:KK174, and 50 nM SHH for 20 hours) in *Smo*^−/−^ cells stably expressing mSMO-WT, mSMO-D99A, mSMO-D477G, or mSMO-V333F. (**E**) A ligand-affinity assay was used to measure the amount of hSMOΔC-BRIL-V329F (*7*) captured on 20(S)-yne–coupled beads in the presence of 50 μM MβCD:20(S)-OHC or MβCD:KK174. The immunoblot shows 1% of the protein added to each binding reaction (input) and 50% of the protein captured on beads. Exact *P* values for comparisons: (C) WT untreated versus KK174 = 0.0003, D99A untreated versus KK174 = 0.0009, and WT versus D99A (+KK174) = 0.9194. (D) WT untreated versus SHH < 0.0001, WT untreated versus SAG < 0.0001, WT untreated versus KK174 = 0.0003, D477G untreated versus SHH < 0.0001, D477G untreated versus SAG > 0.9999, D477G untreated versus KK174 = 0.0002, and WT versus D477G (+KK174) > 0.9999. (F) WT untreated versus SAG < 0.0001, WT untreated versus KK174 < 0.0001, V333F untreated versus SAG > 0.9999, and V333F untreated versus KK174 > 0.9999.

To disentangle the contributions of the CRD and TMD sites using a different strategy, we identified a sterol analog capable of binding and activating SMO selectively through its TMD site. Oxysterols such as 20(S)-OHC are selective CRD agonists, so we reasoned that other sterol analogs may show a preference for the TMD site (*27*–*29*). We focused on KK174, which contains diazirine and alkyne groups on the iso-octyl chain of cholesterol (Fig. 4A) (*30*).

To measure the direct effect of KK174 on SMO in a purified system, we established a pull-down assay to measure the interaction between purified hSMOΔC and the active-state selective Nb8 (Fig. 4B). In the absence of any ligands, hSMOΔC was poorly captured by Nb8-coupled beads. SAG or the CRD-selective agonist 20(S)-OHC promoted the interaction between hSMOΔC and Nb8. Thus, a CRD agonist in a purified system can activate SMO without the need to supply an additional TMD agonist. KK174 also promoted the interaction between hSMOΔC and Nb8, showing that it can directly activate SMO.

KK174 met several criteria for a TMD-selective agonist. Well-established inactivating mutations in the CRD site (D99A) or the SAG site (D477G) failed to impair responses to KK174 (Fig. 4, C and D), showing that it does not activate SMO through the CRD or the SAG sites. This conclusion is also supported by the observation that KK174 does not compete for binding of SMO to 20(S)-yne–coupled beads (Fig. 4E), showing that KK174 does not bind to the CRD (*28*). In addition, the V333F mutation previously used to obstruct the TMD site blocked the signaling response to KK174 (Fig. 4F), showing that KK174 requires the integrity of the TMD site (*13*).

To probe the activity of KK174 on the isolated TMD, we used mSMO-ΔCRD. KK174 could activate mSMO-ΔCRD but not mSMO-ΔCRD-Y398F, demonstrating that Y398 in the TMD is required for KK174 activity (as it is for cholesterol activity) (fig. S5B). KK174 could activate signaling in cholesterol-depleted cells expressing mSMO-ΔCRD, showing that it can mimic cholesterol (fig. S5C).

**Fig. 5.**
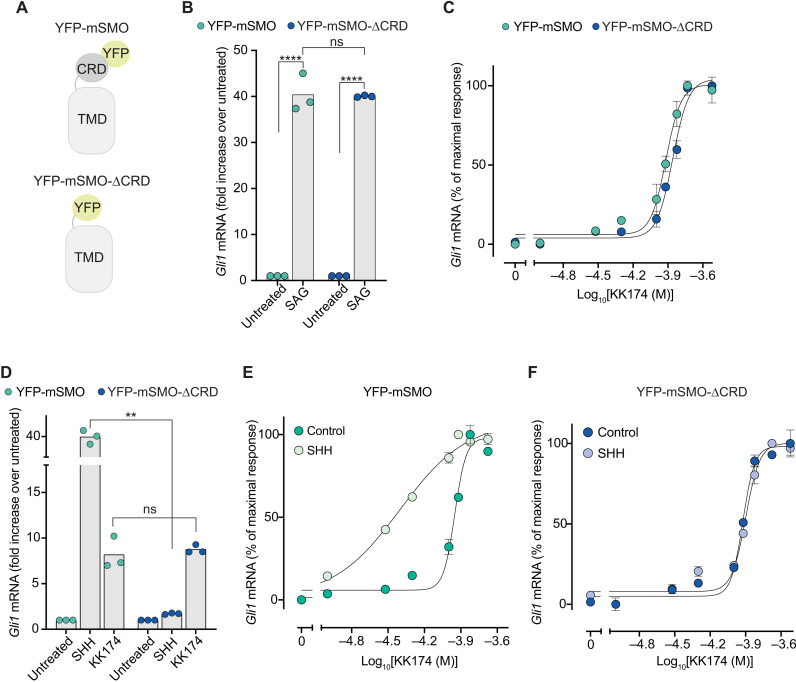
The CRD is required for SMO activation in response to SHH. (**A**) Cartoons showing the domain structures of the YFP-mSMO and YFP-mSMO-ΔCRD proteins used in this figure. (**B**) Fold increase in *Gli1* mRNA induced by SAG (100 nM) in *Smo*^−/−^ cells stably expressing YFP-mSMO or YFP-mSMO-ΔCRD. (**C**) Dose-response curve for KK174 in *Smo*^−/−^ cells stably expressing YFP-mSMO or YFP-mSMO-ΔCRD. On the basis of the curve fits, the EC_50_ of MβCD:KK174 is ~100 μM. (**D**) Fold increase in *Gli1* mRNA induced by SHH (50 nM) or MβCD:KK174 (300 μM) in *Smo*^−/−^ cells stably expressing YFP-mSMO or YFP-mSMO-ΔCRD. (**E** and **F**) Dose-response curves for KK174 in cells expressing either YFP-mSMO or YFP-mSMO-ΔCRD in the presence of a low, subactivating concentration (1 nM) of SHH. In (B) to (F), cells were treated with drugs for 20 hours. Exact *P* values for comparisons: (B) YFP-mSMO untreated versus SAG < 0.0001, YFP-mSMO versus YFP-mSMO-ΔCRD (+SAG) = 0.9957, and YFP-mSMO-ΔCRD untreated versus SAG < 0.0001. (D) YFP-mSMO versus YFP-mSMO-ΔCRD (+SHH) = 0.0025 and YFP-mSMO versus YFP-mSMO-ΔCRD (+KK174) = 0.6355.

Unlike cholesterol, which binds to both the CRD and TMD sites, KK174 allowed us to selectively probe the TMD-binding site in SMO. We took advantage of our previous observation that mSMO-ΔCRD, when fused to yellow fluorescent protein at its N terminus (YFP-mSMO-ΔCRD; Fig. 5A) and stably expressed in *Smo*^−/−^ cells, shows very low basal signaling activity (comparable to that of YFP-tagged mSMO-WT) (*28*). The complete elimination of the CRD in YFP-mSMO-ΔCRD allows unequivocal evaluation of the role of the TMD-site in SMO activation. Both YFP-mSMO and YFP-mSMO-ΔCRD (Fig. 5A) were equally responsive to SAG, demonstrating equivalent signaling capacity (Fig. 5B). KK174 also activated both proteins with similar EC_50_ values (Fig. 5C). These data establish the integrity of the SAG and TMD sites in YFP-mSMO-ΔCRD: Ligands that engage each of these sites retain their agonist properties. By extension, if the SMO TMD site is the primary focus of PTCH1 regulation, then YFP-mSMO-ΔCRD should remain as responsive to SHH as YFP-mSMO. Contrary to this prediction, however, YFP-mSMO-ΔCRD was markedly less responsive to SHH (Fig. 5D), highlighting the essential requirement of the CRD for SHH-triggered signaling. These results also show that KK174 does not act on PTCH1 because in this scenario, the effect of KK174 and SHH on *Gli1* mRNA induction should be similar.

If SHH and KK174 regulate different sites on SMO, then we would predict an additive or synergistic effect on signaling if both ligands are applied simultaneously. If SHH and KK174 both regulate the TMD, then these molecules should show a competitive interaction. Consistent with our previous discovery of an allosteric interaction between the CRD and TMD sites (Fig. 2, D and F), SHH increased the potency (or decreased the EC_50_) of KK174 in cells expressing YFP-mSMO (Fig. 5E). Deletion of the CRD eliminated the potentiating effect of SHH on mSMO activation by KK174, consistent with SHH (and PTCH1) regulating mSMO through the CRD (Fig. 5F). Together, an orthogonal experimental strategy again supported the conclusion that the primary binding site on SMO that is regulated by PTCH1 is the CRD.

### PTCH1 inhibits sterol binding to the SMO CRD

**Fig. 6.**
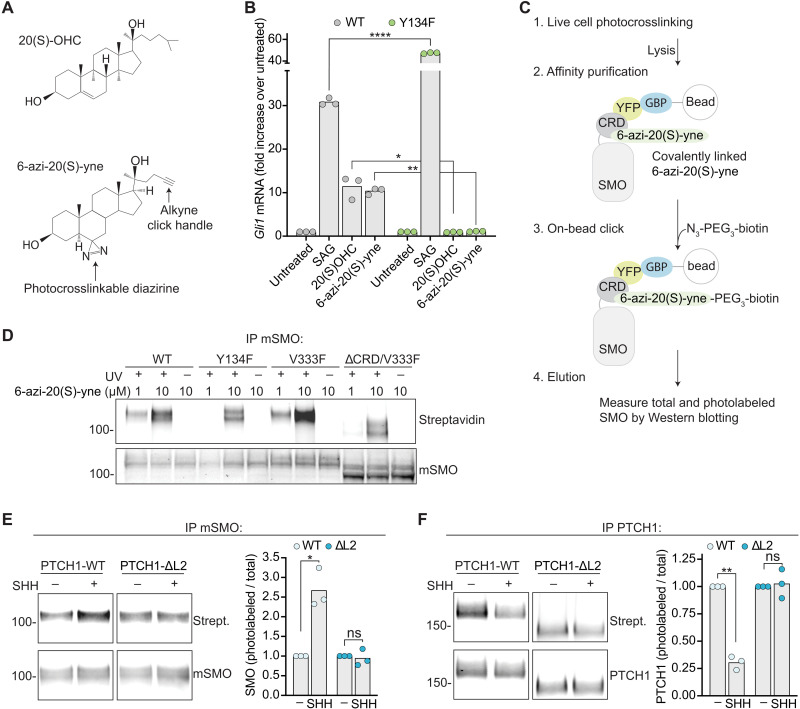
PTCH1 controls sterol binding to the SMO CRD. (**A**) Structures of 20(S)-OHC and 6-azi-20(S)-yne. (**B**) Fold increase in *Gli1* mRNA in response to a 20-hour exposure to 100 nM SAG, 5 μM 20(S)-OHC, or 5 μM 6-azi-20(S)-yne in *Smo*^−/−^ cells stably expressing either mSMO-WT or mSMO-Y134F (*7*). (**C**) Covalent attachment of 6-azi-20(S)-yne to YFP-mSMO in intact cells was measured using diazirine photocrosslinking and click chemistry (*31*). (**D**) Photocrosslinking of 6-azi-20(S)-yne to the indicated YFP-mSMO variants with or without exposure to 365 nm ultraviolet (UV) light. The abundances of photolabeled YFP-mSMO (top) and total YFP-mSMO (mSMO) eluted from beads were measured using immunoblotting (fig. S6B shows inputs). IP, immunoprecipitation. (**E** and **F**) Photocrosslinking of YFP-mSMO (E) or PTCH1 (F) to 6-azi-20(S)-yne was assessed using the pipeline shown in (C). Graphs depict the ratios of photolabeled to total eluted YFP-mSMO or PTCH1 in three independent experiments. Figure S6 (C and D) show inputs, and fig. S6E shows 6-azi-20(S)-yne cross-linking to YFP-mSMO in the presence or absence of PTCH1. Exact *P* values for comparisons: (B) mSMO-WT versus mSMO-Y134F (+SAG) < 0.0001, mSMO-WT versus mSMO-Y134F [+20(S)-OHC] = 0.0208, and mSMO-WT versus mSMO-Y134F [+6-azi-20(S)-yne] = 0.0020. (E) PTCH1-WT untreated versus SHH = 0.0288 and PTCH1-DL2 untreated versus SHH = 0.6955. (F) PTCH1-WT untreated versus SHH = 0.0026 and PTCH1-ΔL2 untreated versus SHH = 0.7730.

If PTCH1 inhibits SMO by preventing sterol access to the CRD, then SHH-mediated inactivation of PTCH1 should lead to enhanced binding of sterols to the CRD. To test this prediction, we developed the oxysterol analog 6-azi-20(S)-yne (Fig. 6A) to measure sterol-CRD interactions in the native membrane environment of intact cells using photoaffinity labeling. This bifunctional analog is decorated with a terminal alkyne compatible with click chemistry and a photocrosslinkable diazirine. 6-azi-20(S)-yne activated Hh signaling in a manner similar to 20(S)-OHC (Fig. 6B) with an EC_50_ of ~0.5 μM (fig. S6A). 6-azi-20(S)-yne activates mSMO through its CRD because a point mutation (Y134F; fig. S2A) in the CRD site abrogated 6-azi-20(S)-yne–mediated signaling (Fig. 6B). To measure the interaction of YFP-mSMO with 6-azi-20(S)-yne, live cells were irradiated with 365 nm light to activate the diazirine and initiate covalent labeling of interacting proteins (*31*). YFP-mSMO was then immunopurified from detergent extracts, and on-bead click chemistry was used to attach a biotin to the YFP-mSMO-sterol adduct (Fig. 6C). Western blotting with streptavidin was used to measure the amount of YFP-mSMO photo-labeled by 6-azi-20(S)-yne.

To test the specificity of CRD photolabeling by 6-azi-20(S)-yne, we used YFP-mSMO-WT and YFP-mSMO variants carrying mutations in the CRD site (Y134F) or the TMD site (V333F). When used at a concentration of 1 μM, 6-azi-20(S)-yne demonstrated specific cross-linking to YFP-mSMO variants with an intact CRD (Fig. 6D): YFP-mSMO-Y134F was not labeled, while YFP-mSMO-V333F was labeled at a similar efficiency as YFP-mSMO-WT. Photolabeling of YFP-mSMO-V333F was abolished when the CRD was deleted (Fig. 6D). As expected, the photolabeling of YFP-mSMO was strictly dependent on ultraviolet (UV) irradiation. All experiments used a concentration of 1 μM 6-azi-20(S)-yne, since nonspecific photolabeling was observed at 10 μM (Fig. 6D).

We measured YFP-mSMO-WT photolabeling by 6-azi-20(S)-yne in the presence of PTCH1-WT or a widely used deletion mutant of PTCH1 (PTCH1-ΔL2). PTCH1-ΔL2 can inhibit SMO activity but cannot bind or be inactivated by SHH (*32*, *33*). The addition of SHH increased YFP-mSMO-WT photolabeling by 6-azi-20(S)-yne in cells expressing PTCH1-WT but failed to change YFP-mSMO-WT photolabeling in cells expressing the SHH-unresponsive mutant PTCH1-ΔL2 (Fig. 6E). These data suggest that PTCH1 can reduce the access of the SMO CRD to 6-azi-20(S)-yne. PTCH1 itself could be photolabeled by 6-azi-20(S)-yne. Labeling of PTCH1 by 6-azi-20(S)-yne was diminished by SHH, an inhibitor of PTCH1 transport activity (Fig. 6F). As predicted, photolabeling of PTCH1-ΔL2 was not blocked by SHH.

In summary, our data provide direct support for the model that PTCH1 uses its transport activity to regulate SMO by reducing the binding of a sterol to its CRD. Our results are in agreement with prior work showing that SHH and PTCH1 can regulate SMO labeling by a cholesterol analog that undergoes spontaneous (not UV-catalyzed) covalent attachment to the CRD (*12*).

## DISCUSSION

Despite a bounty of structural studies, the mechanism by which sterols activate SMO remains uncertain. The presence of multiple ligand-binding sites in SMO raises the question of which site is the orthosteric site (or the primary focus of regulation by PTCH1) and which site functions as an allosteric site. While GPCRs can be regulated by multiple ligands at orthosteric and allosteric sites, SMO is unusual in two ways. First, both the TMD and CRD sites of SMO engage the same ligand, cholesterol. Second, the high abundance of cholesterol in the plasma membrane of vertebrate cells means that SMO likely never exists in a truly “ligand-free” state (*34*, *35*). This is particularly true of the TMD, positioned within the interior of the membrane bilayer where cholesterol represents one of every three lipid molecules. Approximately 20 molecules of cholesterol are predicted to be present in the lipid layer immediately surrounding the TMD (*3*). For example, simulation studies provide support for cholesterol molecules located between helices α2 and α3 (*16*). In contrast, the CRD site is positioned >10 Å above the plane of the plasma membrane (*5*, *7*). Access to the CRD presents a substantial energetic barrier because it requires a hydrophobic cholesterol molecule to completely desorb from the membrane and traverse an aqueous environment (*36*). These considerations suggest that the CRD site is better suited to be regulated by PTCH1 than the cholesterol-immersed TMD site, at least in vertebrate plasma membranes that contain 30 to 50 mole percent (mol %) of cholesterol. The TMD site may play a more central role in PTCH1 regulation in insects, whose membranes have markedly lower amounts of sterols (<5 mol %; most of which is ergosterol) (*37*, *38*). The CRD from *Drosophila* SMO does not bind oxysterols in vitro (*28*, *39*).

**Fig. 7.**
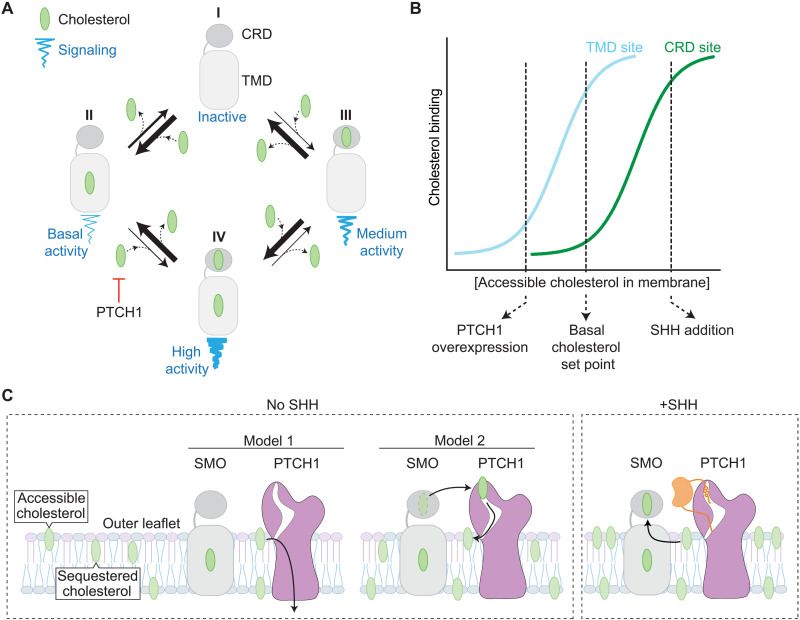
A model for SMO activation by dual sterol binding to the TMD and CRD sites. (**A**) Linked equilibria showing four states of SMO defined by sterol occupancy of the CRD and TMD sites. At physiological membrane cholesterol levels, SMO mostly exists in state II, with cholesterol bound to the TMD driving basal signaling activity. PTCH1 inhibits the transition from state II to the fully active state IV by reducing accessible cholesterol in the outer leaflet of the membrane. (**B**) A cartoon depicting the binding of cholesterol to the TMD site (cyan) or the CRD site (green) at increasing levels of accessible cholesterol in the membrane outer leaflet. Under basal (no SHH) conditions, the TMD site is largely occupied, while the CRD site is largely empty. PTCH1 overexpression (especially above physiological levels) can reduce cholesterol binding to both sites by depleting the membrane outer leaflet of accessible cholesterol. Conversely, SHH increases cholesterol binding to the CRD by inactivating PTCH1. (**C**) Two models for the regulation of SMO signaling by PTCH1. In model 1, PTCH1 depletes the membrane of accessible cholesterol, indirectly inhibiting SMO, while in model 2, PTCH1 directly removes cholesterol from the SMO CRD.

On the basis of three different lines of investigation, we propose a model for SMO activation (Fig. 7A) that considers four states of SMO with varying signaling activities. When both the CRD and TMD sites are unoccupied, SMO has minimal activity (as seen for SMO-D99A/Y398F; Fig. 2D). When both the CRD and TMD sites are occupied by cholesterol in response to SHH, SMO has maximal signaling activity. Two additional states are defined by sterol binding to either the CRD or the TMD site, with the other remaining empty. In the absence of SHH, the abundance of cholesterol in the membrane leads to occupancy of the TMD site, driving basal signaling activity. However, PTCH1 uses its transporter activity to reduce accessible cholesterol in the outer leaflet of the ciliary membrane (*2*, *4*), ensuring that the CRD site remains unoccupied and, consequently, the CRD maintains its inhibitory restraint on the TMD. When PTCH1 is inactivated by SHH, cholesterol binds to the CRD because the concentration of accessible cholesterol in the outer leaflet of the membrane rises (*4*), driving full SMO activation. Both the CRD and TMD sites do not have to be occupied for SMO activation: The SHH-induced fold increase in SMO activity is maintained even when the TMD site is mutated (e.g., in mSMO-Y398F). On the other hand, basal signaling activity (in the absence of SHH) is driven largely by cholesterol binding to the TMD even in the presence of CRD mutations (Fig. 3).

A key observation that emerged from our experiments is that mutation of the SMO TMD site reduces both the basal and SHH-induced levels of SMO signaling but leaves the fold increase (the ratio of SHH-induced to basal activity) intact. Our results are in agreement with previous data showing that mutations in the TMD site reduce absolute levels of SMO signaling, a result that was used to argue that the TMD site is the PTCH1-regulated orthosteric site in SMO (*13*). However, our observation that the SHH-induced fold increase in SMO activity is unchanged by mutations in the TMD site is more consistent with its role as an allosteric site regulating absolute SMO signaling activity across the range of SHH concentrations. Our data show that the fold change in SMO signaling activity in response to SHH is mediated primarily by the orthosteric CRD site.

The most compelling evidence that the TMD site is the primary focus of PTCH1 regulation is the observation that the signaling activity of SMO carrying mutations in the CRD is still partially sensitive to PTCH1 (*17*, *27*). For example, the basal signaling activity of SMO-ΔCRD (lacking the CRD) can be suppressed by overexpression of PTCH1 (*27*). The common use of cholesterol as both an allosteric and orthosteric agonist of SMO provides an explanation for these previous results. Because PTCH1 reduces cholesterol accessibility in the membrane, it can reduce cholesterol binding to both the CRD and TMD sites, especially when PTCH1 is overexpressed at high, nonphysiological levels used in these previous studies (Fig. 7B). The ability of sterols to alter SMO activity by binding to the allosteric TMD site is not unexpected—ligands that bind to allosteric sites on GPCRs can function as agonists in their own right and change receptor signaling activity (*40*). However, our results show that under endogenous expression levels, PTCH1 primarily suppresses SMO activity by preventing cholesterol occupancy of the CRD cholesterol–binding site. Notably, PTCH1 is an immediate-early Hh target gene, and its induction in response to SHH is a major negative-feedback loop in the pathway. In tissues exposed to high SHH concentrations, it remains possible that the high abundance of PTCH1 reduces cholesterol binding to both the CRD and the TMD sites (Fig. 7B).

A recently proposed third model for SMO activation by sterols suggests that there is continuous movement of sterols along a tunnel that extends from the TMD to the CRD in SMO (*15*). SMO activation is triggered by sterol trapping in the TMD site, perhaps because all the sterol-binding sites in this tunnel are occupied when membrane sterol levels rise. Our results contradict this model for two reasons. First, SHH can increase the activity of mSMO carrying a mutation in the TMD site (mSMO-Y398F) by the same extent as mSMO-WT. Second, YFP-mSMO-ΔCRD is poorly activated by SHH although it remains normally responsive to both SAG and the TMD-agonist KK174.

Most models for how PTCH1 inhibits SMO converge around the concept that PTCH1, using its transporter function, reduces the abundance of a sterol activator of SMO (*1*, *3*, *41*). Our observation that the same sterol, 6-azi-20(S)-yne, interacts with both PTCH1 and SMO in an SHH-regulated manner supports a key tenet of this model: The sterol activator of SMO should also be a substrate for PTCH1. The photocrosslinking of 6-azi-20(S)-yne to PTCH1 can be reduced by SHH, suggesting that it is a PTCH1 substrate, and inactivation of PTCH1 by SHH increases the photocrosslinking of 6-azi-20(S)-yne to SMO. These results have interesting implications for how PTCH1 inactivates SMO. Two distinct (but not mutually exclusive) models for PTCH1-SMO regulation are as follows: (i) PTCH1 reduces the cholesterol accessibility in the membrane compartment where SMO signals or (ii) PTCH1 directly inactivates SMO using its extracellular domains to remove cholesterol from the SMO CRD (Fig. 7C) (*1*, *2*, *42*, *43*). Unlike cholesterol, oxysterols such as 6-azi-20(S)-yne do not accumulate in membranes because they carry a second hydrophilic hydroxyl group on the iso-octyl chain (*44*). The observation that PTCH1 reduces 6-azi-20(S)-yne cross-linking to SMO suggests that it may also function by directly removing sterols from the SMO CRD (Fig. 7C).

## MATERIALS AND METHODS

### Constructs and plasmids

#### *SMO constructs*

Full-length untagged mSMO mutants (D99A, Y134F, D99A/Y134F, Y398F, D99A/Y398F, V333F, and D477G) were generated using the QuikChange method. Amino acid residues 67 to 184 were deleted from mSMO to generate mSMO-ΔCRD, and point mutants (ΔCRD-Y398F, ΔCRD-V333F) were introduced into the mSMO-ΔCRD backbone. YFP-tagged SMO constructs (YFP-mSMO and YFP-mSMO-ΔCRD) were generated by introducing the YFP tag downstream of the mSMO signal sequence, as described previously (*45*). All constructs were cloned into the pMSCVpuro vector to enable retrovirus generation for stable cell line construction.

For the Nb8 binding studies shown in Figs. 3 and 4, variants of the hSMO protein lacking its disordered C-terminal cytoplasmic tail were used: hSMOΔC (containing amino acid residues 32 to 555, UniProt ID Q99835), hSMOΔC-ΔCRD (containing amino acid residues 190 to 555), and hSMOΔC-BRIL-V329F (which contains a BRIL domain inserted in place of intracellular loop 3 and the V329F-inactivating point mutation). hSMOΔC constructs were C-terminally tagged with 1D4 and N-terminally tagged with a hemagglutinin (HA) tag.

#### *Nb8 constructs*

A synthetic gene for the active-conformation specific nanobody Nb8 (*13*) was obtained from GeneArt (Thermo Fisher Scientific) and cloned into pHR-CMV-TetO2 to generate a fusion at its C terminus to a HRV3C-mVenus-12His fragment.

#### *PTCH1 constructs*

Full-length PTCH1-WT and PTCH1-ΔL2 were fused to a C-terminal 1D4 tag (amino acid sequence: TETSQVAPA) and cloned into the pcDNA5-FRT-TO Flp-In vector (Thermo Fisher Scientific, catalog no. V652020) to enable inducible expression in the 293Trex Flp-In cell system. PTCH1-ΔL2, which carries a deletion of the second extracellular loop (L2, amino acids 793 to 994), was a gift from J. Briscoe (*32*).

### Cell lines

Human embryonic kidney (HEK) 293S GnTI cells [American Type Culture Collection (ATCC), catalog no. CLR-3022], HEK293T cells (ATCC, catalog no. CRL-3216), and Lenti-X cells (Takara Bio, catalog no. 632180) were purchased and used at low passages without further authentication. *Smo*^−/−^ mouse embryonic fibroblasts (MEFs) used to stably express SMO variants have been described previously (*46*) and were tested to ensure lack of endogenous SMO protein using immunoblotting. All stable cell lines derived from *Smo*^−/−^ cells were generated as described previously (*28*) and authenticated by immunoblotting to ensure stable expression of the transgene. Cell lines were confirmed to be negative for mycoplasma infection. Stable 293Trex Flp-In cells (Thermo Fisher Scientific, catalog no. R780-07) expressing PTCH1 were generated using PTCH1 constructs cloned into the pcDNA5 FRT-TO Flp-In vector according to the manufacturer’s instructions (*4*).

### SMO expression and purification for crystallization and binding studies

hSMOΔC-BRIL-V329F and its variants were expressed and purified as reported previously (*7*). For other SMO proteins, constructs were cloned into pHR-CMV-TetO2 (Addgene plasmid no. 113883) with an added 3C-protease cleavable mVenus-1D4 tag for generation of inducible stable HEK293S GnTI cell lines for expression (*47*).

Stable cell lines were expanded in Dulbecco’s modified Eagle’s medium (DMEM; Sigma-Aldrich) supplemented with 10% fetal bovine serum (FBS; Gibco), 1% l-glutamine (Gibco), and 1% nonessential amino acids (NEAAs; Gibco). Cells were transferred to Freestyle 293 expression medium (Gibco) (supplemented with 1% FBS, 1% l-glutamine, and 1% NEAAs) and grown in suspension format at 37°C and 8% CO_2_ with 130 rpm shaking until a cellular density of 3 × 10^6^ to 4 × 10^6^/ml was reached. Expression was induced with doxycycline (0.1 μg/ml; Sigma-Aldrich), and cells were harvested after 72 hours by centrifugation (1500*g* for 10 min). Cell pellets were resuspended in chilled 50 mM Hepes (pH 7.5), 300 mM NaCl, and 1% protease inhibitor cocktail (Sigma-Aldrich, P8340). This was supplemented with a final concentration of 1% 2,2-dioctylpropane-1,3-bis-β-d-maltopyranoside (DMNG; Anatrace) and 0.1% cholesteryl hemisuccinate (CHS; Anatrace) and incubated at 4°C for 90 min. Cell lysate was clarified by centrifugation (50,000*g* for 30 min at 4°C), and the soluble fraction was retained. A total of 50 mM Hepes (pH 7.5) and 300 mM NaCl were added to dilute the total detergent concentration to 0.5% before adding Rho-1D4 antibody–coupled (University of British Columbia) CNBr-activated sepharose beads (Cytiva) and incubating for 2 hours. Protein-bound beads were washed sequentially with 50 mM Hepes (pH 7.5), 300 mM NaCl, 10% glycerol, 0.007% DMNG, and 0.0007% CHS, followed by 50 mM Hepes (pH 7.5), 300 mM NaCl, 10% glycerol, 0.06% glyco-diosgenin (GDN; Anatrace), and 0.006% CHS, and last, with 50 mM Hepes (pH 7.5), 300 mM NaCl, 10% glycerol, 0.02% GDN (Anatrace), and 0.002% CHS. Protein was eluted overnight with the addition of 3C protease (produced in-house) and concentrated to 250 μl using a Vivaspin 20 polyethersulfone 100,000-Da molecular weight cutoff centrifugal concentrator (Sartorius). Protein was loaded onto a Superose 6 Increase 10/300 column (Cytiva) preequilibrated in 10 mM Hepes (pH 7.5), 150 mM NaCl, 10% glycerol, 0.02% GDN, and 0.002% CHS. Peak fractions were pooled, concentrated, and frozen.

### Nb8-mVenus expression and purification

HEK293T Lenti-X cells were used to generate lentiviruses using the pHR-CMV-TetO2 vector and subsequently used to infect HEK293T cells for expression (*47*). Stable HEK293 T cells were expanded into roller bottles in DMEM (Sigma-Aldrich, catalog no. D6546) supplemented with 1% l-glutamine, 1% NEAAs, and 10% FBS. To induce expression, the medium was switched to 2% FBS and incubated at 37°C and 5% CO_2_ for 72 hours. Conditioned medium was harvested and replaced with fresh complete DMEM supplemented with 2% FBS and incubated for a further 72 hours before the final harvest. Cell debris was removed from conditioned medium by centrifugation (4000*g* for 10 min at room temperature) and filtration (0.2 μM). Clarified medium was concentrated and dialyzed into phosphate-buffered saline (PBS; pH 8.0) using a QuixStand benchtop system (Cytiva) connected to a 60-cm Xampler Cartridge (Cytiva) with a 10-kDa molecular weight cutoff. Concentrated medium was incubated with TALON beads for 1 hour at room temperature. Protein-bound beads were washed sequentially with PBS (pH 8.0) and PBS (pH 8.0) supplemented with 5 mM imidazole. Protein was eluted in 10 mM Hepes (pH 7.5), 150 mM NaCl, and 250 mM imidazole and loaded onto a Superdex75 16/600, preequilibrated in 10 mM Hepes (pH 7.5) and 150 mM NaCl. Peak fractions were pooled and concentrated, and aliquots were snap-frozen in liquid nitrogen for storage at −80°C.

### Nb8 FSEC binding assay

Samples were prepared using purified hSMOΔC variants and Nb8-mVenus in reaction volumes of 200 μl and incubated for 1 or 24 hours. A total of 10 μl was loaded onto a Superose 6 Increase 3.2/300 column (Cytiva) preequilibrated in 10 mM Hepes (pH 7.5), 150 mM NaCl, 0.02% GDN, and 0.002% CHS. Absorbance at 280 nm was monitored, and mVenus fluorescence was measured using in-line fluorescence detection (excitation, 515 nm; emission, 528 nm).

### Nb8 bead binding assay

Purified hSMOΔC was incubated overnight at 4°C with an excess of Nb8 and small molecule ligands (100 μM each), where applicable. Nb8 was immobilized onto TALON beads via its 12-His tag and washed with 10× bead volumes of chilled 10 mM Hepes (pH 7.5), 150 mM NaCl, 0.02% GDN, and 0.002% CHS. Nb8 and any bound SMO were eluted in 10 mM Hepes (pH 7.5), 150 mM NaCl, 0.02% GDN, 0.002% CHS, and 250 mM imidazole and analyzed by Western blotting using the HA (monoclonal, clone 2-2.2.14, Thermo Fisher Scientific, catalog no. 26183; Research Resource Identifier (RRID): AB_2533056) tag appended to hSMO∆C.

### Crystallization and data collection

SAG (EMD Millipore, SAG1.3) dissolved at high concentration in dimethyl sulfoxide (DMSO) was added directly to the purified hSMOΔC-BRIL-V329F protein after deglycosylation. The final concentration of SAG and its approximate molar excess with respect to purified SMO at ~30 mg/ml (~465 μM) was 8.35 mM SAG corresponding to an 18× molar excess. This resulted in a protein-SAG solution with a final concentration of 2.5% DMSO. The hSMOΔC-BRIL-V329F:SAG complex was reconstituted into lipidic cubic phase by mixing with molten lipid in a mechanical syringe mixer (*48*). Lipid, consisting of 10% cholesterol (Sigma-Aldrich) and 90% 9.9 monoacylglycerol (monoolein, Sigma-Aldrich), was heated to 42°C before mixing with detergent-solubilized protein (with small molecule added) at ~30 mg/ml in a ratio of 3:2 (w/v). A Gryphon robot (Art Robbins Instruments) was used to dispense 50 nl boluses of protein-laden mesophase followed by 0.8 μl of precipitant solution onto each of 96 positions on a siliconized glass plate and then sealed with a glass coverslip in a “sandwich-plate” format. The hSMOΔC-BRILV329F: SAG complex crystallized in 0.1 M MES (pH 6.0), 0.09 to 0.12 M potassium formate, 24 to 27% (v/v) polyethylene glycol dimethyl ether 500, 0.5 mM zinc chloride, and 0.1 M ammonium fluoride. Crystals were grown at 20°C and monitored by eye, using a microscope fitted with cross-polarizers. Data collection details are provided in table S1.

### Structure determination, refinement, and analysis

X-ray data were processed using the automated processing software Xia2 (*49*). Typical individual hSMOΔC-BRIL-V329F crystals generated only 20° to 45° wedges of usable data due to radiation damage. For the hSMOΔC-BRIL-V329F:SAG dataset, three such wedge datasets were merged. Data collection and processing statistics are shown in table S1. The hSMOΔC-BRIL-V329F:SAG complex structure was solved by molecular replacement in PHASER (*50*) using the structure of hSMOΔC-BRIL-V329F [Protein Data Bank (PDB) 5L7D] (*7*) as the search model. The asymmetric unit of each complex contained two copies of SMO in the same antiparallel arrangement observed previously (*7*). The structures were refined using BUSTER (*51*) and PHENIX (*52*) using noncrystallographic and secondary structure restraints. Extra electron density accounting for SAG, cholesterol, a n-acetylglucosamine (NAG) moiety attached to N188 in the connector region and one well-ordered monoolein molecule was apparent after molecular replacement. Refinement statistics are provided in table S1.

### Cell culture and drug treatments for Hh signaling assays

All cells were grown in high-glucose DMEM (Thermo Fisher Scientific, catalog no. SH30081FS) containing 10% FBS (Sigma-Aldrich, catalog no. S11150) and the following supplements (hereafter called supplemented DMEM): 1 mM sodium pyruvate (Gibco, catalog no. 11-360-070), 2 mM l-glutamine (GeminiBio, catalog no. 400106), 1× minimum essential medium NEAA solution (Gibco, catalog no. 11140076), penicillin (40 U/ml), and streptomycin (40 μg/ml) (GeminiBio, catalog no. 400109). Supplemented DMEM was sterilized through a 0.2-μm filter and stored at 4°C.

To deplete cells of cholesterol in Fig. 3C, cells were seeded and grown in supplemented DMEM containing 5% lipoprotein-depleted serum (LDS) (Kalen Biomedical LLC) in place of 10% FBS. After 1 week of growth, cells were seeded for experiments in fresh 5% LDS-supplemented DMEM and grown until confluent. Cells were then serum-starved in 0.5% LDS-supplemented DMEM and simultaneously treated with 1 μM U18666A (Sigma-Aldrich) for 20 hours before the experiment. To rescue cholesterol depletion, 10 μM cholesterol was delivered as methyl-b-cyclodextrin (MbCD):cholesterol complexes at time of serum starvation [generation of MβCD:cholesterol complexes is described in (*5*)].

To measure Hh responsiveness either by quantitative polymerase chain reaction (PCR) or Western blot, cells were seeded in 10% FBS-supplemented DMEM and grown to confluence. To induce ciliation, cells were then serum-starved in 0.5% FBS-supplemented DMEM and simultaneously treated. All treatment times and agonist/antagonist concentrations are supplied in the figure legends. MβCD:KK174 complexes were generated by the same method used to generate MβCD:cholesterol (*5*), where the MβCD:cholesterol ratio is ~8.8:1, and concentrations reported reflect the MβCD concentration due to their higher accuracy.

### Hh signaling assays

#### *Quantitative PCR*

A method to analyze *Gli1* mRNA transcript levels was described previously (*2*).

#### *Immunoblotting*

Whole-cell extracts were prepared in lysis buffer containing 150 mM NaCl, 50 mM tris-HCl (pH 8), 10% NP-40, 1× protease inhibitor (SigmaFast Protease inhibitor cocktail, EDTA-free; Sigma-Aldrich, catalog no. S8830), 1 mM MgCl_2_, and 10% glycerol. After lysate clarification by centrifugation at 20,000*g*, samples were resuspended in 50 mM tris(2-carboxyethyl)phosphine and 1× Laemmli buffer for 30 min at 37°C. Samples were then subjected to SDS–polyacrylamide gel electrophoresis, followed by immunoblotting with antibodies against GLI1 [anti-GLI1 mouse monoclonal (clone L42B10); Cell Signaling Technology, catalog no. 2643, RRID: AB_2294746], SMO (rabbit polyclonal) (*46*), P38 (anti-P38 rabbit polyclonal; Abcam, catalog no. ab7952; RRID: AB_306166), or a-tubulin [anti–a-TUBULIN (clone DM1A), MilliporeSigma, catalog no. T6199, RR1D: AB_477583].

### Detection of SMO at cilia using immunofluorescence

*Smo^−/−^* MEFs stably expressing SMO variants were seeded on acid-washed coverslips and grown to confluency. The medium was then exchanged for 0.5% FBS-supplemented DMEM to induce ciliation, and cells were treated with indicated treatments. Around 16 to 24 hours later, cells were fixed, blocked, and permeabilized in blocking buffer [0.1% Triton X-100, 1% donkey serum, bovine serum albumin (10 mg/ml), and PBS]. Primary antibodies against SMO (rabbit anti-SMO) (*46*) and ARL13B to detect primary cilia (guinea pig anti-ARL13B) (*53*) were then added. After washing with 0.1% Triton X-100/PBS, secondary antibodies were added [donkey anti-rabbit immunoglobulin G (IgG) (H + L) highly cross-adsorbed secondary antibody, Alexa Fluor 488, Thermo Fisher Scientific (Invitrogen, catalog no. A-21206, RRID: AB_2535792); and Alexa Fluor 647 AffiniPure donkey anti-guinea pig IgG (H + L) (Jackson ImmunoResearch Laboratories, catalog no. 706-605-148, RRID: AB_2340476)]. Cells were then washed and mounted on glass slides. Images were acquired on an inverted Leica SP8 laser scanning confocal microscope with a 63× oil immersion objective lens (numerical aperture 1.4). Z stacks were acquired with identical acquisition settings (gain, offset, laser power) within a given experiment. Analysis was carried out as previously described (*54*) and analyzed using publically available scripts on GitHub (https://github.com/heybhaven/Cilia_protein_quantification).

### Ligand affinity assay to measure the interaction between SMO and 20(S)-yne

Generation of 20(S)-yne–coupled beads and purification of SMO lacking its C-terminal domain (hSMOΔC-BRIL-V329F) have both been described in detail previously (*7*, *9*). To test for bead binding, purified hSMOΔC-BRIL-V329F diluted in binding buffer [50 mM Hepes (pH 7.5), 300 mM NaCl, 10% glycerol, 0.03% n-Dodecyl-b-D-Maltoside (DDM), and 0.006% CHS] was incubated with oxysterol beads (prewashed with binding buffer) overnight at 4°C in the presence or absence of 50 μM MβCD:20(S)-OHC or MβCD:KK174. Unbound material was removed by washing with binding buffer three times, and the amount of SMO retained on the beads was measured by elution from the beads using Laemmli buffer (30 min at 37°C), followed by Western blotting using the HA (monoclonal, clone 2-2.2.14, Thermo Fisher Scientific, catalog no. 26183; RRID: AB_2533056) tag appended to hSMOΔC-BRIL-V329F.

### Photoaffinity labeling of SMO, followed by click chemistry–based detection

To measure the interaction between 6-azi-20(S)-yne and mSMO in intact cells, 293 T cells (ATCC, catalog no. CRL-3216) were plated in 10% FBS-supplemented DMEM and grown to 75% confluency. The medium was exchanged to 5% LDS-supplemented DMEM (see the “Cell culture and drug treatments for Hh signaling assays” section), and N-terminally YFP-tagged mSMO (YFP-mSMO) was transiently transfected using polyethylenimine. The following day, medium was exchanged to 0.5% LDS-supplemented DMEM containing 1 μM lovastatin, 10 μM lithium mevalonate, and 1 μM U18666A. Twenty hours after YFP-mSMO transfection, 1 μM 6-azi-20(S)-yne was added in the presence or absence of SHH (1 μM) for 1 hour. Cells were then moved to a metal rack on ice, medium exchanged to ice-cold 1× PBS with 1× protease inhibitor cocktail (SigmaFast Protease inhibitor cocktail, EDTA-free; Sigma-Aldrich, catalog no. S8830), and irradiated with 365 nm light for 30 min to trigger photocrosslinking to the diazirine.

After the photolabeling step, cells were immediately lysed for 1 hour at 4°C in lysis buffer [10% DDM, 0.1% SDS, 50 mM Hepes (pH 7.4), 150 mM NaCl, 1× protease inhibitor cocktail, and 10% glycerol], and the resulting lysate was clarified by centrifugation (20,000*g* for 30 min at 4°C). PTCH1 or SMO proteins were immunoprecipitated using antibody-coupled beads (1 hour at 4°C), and the beads were washed five times with wash buffer [10% DDM, 0.1% SDS, 50 mM Hepes (pH 7.4), 150 mM NaCl, and 10% glycerol].

To detect mSMO or PTCH1 photolabeled with 6-azi-20(S)-yne, an on-bead click reaction was performed to attach a biotin via the alkyne handle on 6-azi-20(S)-yne. Beads were resuspended in 100 μl of wash buffer and supplemented with the following reagents in this order: (i) 2.5 μl of freshly prepared 200 mM sodium ascorbate, (ii) 1 μl of 10 mM tris-hydroxypropyltriazolylmethylamine, (iii) 2 μl of 5 mM PEG-biotin-azide, and (iv) 1 μl of freshly prepared 100 mM copper sulfate. After incubation at 37°C for 30 min, the reaction was quenched, and the protein eluted using elution buffer [50 mM Hepes (pH 7.4), 4% SDS, and 5 mM EDTA; 30 min with agitation at room temperature]. The eluate was used for immunoblotting to detect total PTCH1 and mSMO [PTCH1 was detected using an antibody recognizing its 1D4 tag: mouse monoclonal anti-1D4 (The University of British Columbia; RRID: AB_325050); mSMO was detected using the rabbit polyclonal antibody used previously (*46*)]; secondary antibodies included IRDye 680LT donkey anti-mouse IgG (LI-COR Biosciences; RRID: AB_2814906), IRDye 680LT donkey anti-rabbit IgG (LI-COR Biosciences; RRID: AB_2814907), and photolabeled PTCH1 and mSMO using streptavidin reagents (LI-COR IRDye 800CW Streptavidin, LI-COR Biosciences, catalog no. 926-32230). Images were captured on the LI-COR Odyssey Imaging Platform.

### 6-azi-20(S)-yne synthesis 

See Supplementary Methods.

### Constructs used in atomistic molecular dynamics simulations

The activated mSMO structure, bound to CRD and TMD cholesterols, SAG, and Nb8, was obtained from the PDB (PDB 6O3C) (*13*). Extracellular loop 3 (ECL3) was modeled using the mSMO sequence based on an existing hSMOΔC-BRIL-V329F structure (PDB 5L7D) (*7*) using MODELLER9.20 (*55*). PropKa (*56*) was used to identify three residues with nonstandard pKa’s (E485, E522, and H73), which were modeled accordingly. The following systems were constructed for simulation: (i) mSMO bound to the nanobody, both cholesterols and SAG, (ii) mSMO bound to CRD cholesterol and SAG, (iii) mSMO with both CRD and TMD cholesterol bound, (iv) mSMO bound to CRD cholesterol, (v) mSMO bound to TMD cholesterol, and (vi) mSMO in its *apo* form. An additional system of mSMO bound to three cholesterols (with the third cholesterol occupying a “Mid” position in proximity to the linker region) was constructed on the basis of the position of the upper 24(S),25-epoxycholesterol in PDB 6XBM (fig. S3, C to H) (*15*). Systems of hSMO bound to CRD cholesterol and SAG were constructed using the crystal structure in this manuscript (Fig. 1B). The V329F mutant was reverted to WT, the BRIL insert removed, and the intracellular loop 3 (ICL3) and ECL3 were rebuilt on the basis of the position in an existing structure (PDB 4JKV, (*18*)) using MODELLER9.20 (*55*).

Each system was embedded in a 9 nm–by 9 nm 1-palmitoyl-2-oleoyl-sn-glycero-3-phosphocholine (POPC):cholesterol (3:1) bilayer using CHARMM-GUI (*57*). Protein components were described using the AMBER-ff14SB forcefield (*58*). Glycan GlcNAc attachments were modeled on N80 and N497 of mSMO and N188 and N493 of hSMO. Glycans were described using the GLYCAM-06j forcefield with associated amino acid attachment parameters (*59*). SAG parameters were derived using antechamber and the generalized AMBER forcefield (GAFF2) with AM1-BCC used to optimize charge interactions (*60*, *61*). Lipids (including bound cholesterols) were described using the AMBER-lipid17 forcefield (https://ambermd.org/AmberModels_lipids.php). The system was solved using TIP4P-Ew water and approximately 0.15 M NaCl (*62*, *63*).

### Atomistic molecular dynamics simulations

Simulations were performed using GROMACS 2019 (www.gromacs.org). Each system was minimized using a steepest descent method before Number of particles, Volume, Temperature (NVT) and Number of particles, Pressure, Temperature (NPT) equilibration steps of 5 ns each with restraints applied to protein, glycan, and ligand coordinates when present. Atomistic simulations (5 × 300 ns) were performed for each system. A 2-fs timestep was used and periodic boundary conditions applied. Temperature was maintained at 310 K using the Nosé-Hoover thermostat and a τ_T_ = 0.5-ps coupling constant (*64*, *65*). The Parrinello-Rahman barostat with a τ_P_ = 2.0-ps coupling constant and a compressibility of 4.5 × 10^−5^ bar^−1^ was used to maintain pressure at 1 bar (*66*). Electrostatics was described using the particle-mesh Ewald method with a 1.2-nm cutoff. Van der Waals interactions were modeled with a 1.2-nm cutoff using the Verlet method. Long-range dispersion corrections were applied for energy and pressure. All bonds were constrained to the equilibrium lengths using the LINCS algorithm (*67*). Analysis of hydrogen bonds between Y398 and the 3β-hydroxyl group of the TMD cholesterol was calculated using the HydrogenBondAnalysis class of MDAnalysis and a 3-Å cutoff (*68*).

Allosteric pathways were calculated using allopath (github.com/delemottelab/allosteric-pathways) (*22*). Each residue in mSMO was assigned to a node. Source indices were defined as the Cα atoms of residues lining the CRD (D99, L112, W113, Y134, I160, V161, and I500). Sink indices were defined as the Cα atoms of the proposed ionic-lock residues on α6/α7 [R455 (R^6.32^), F459 (F^6.36^), and W539 (W^7.55^)], previously identified as playing a role in the activation of SMO and class F GPCRs (*14*, *69*). The heavy atoms of each cholesterol molecule were considered to comprise a lipid (“interactor”) node. A cutoff value of *c* = 0.45 nm and an SD of σ = 0.138 nm were used to define heavy atom contacts during construction of the contact map, as described previously (*22*). Information flow was calculated in simulations of mSMO initiated from a ligand free *apo* state, with cholesterol bound to the CRD alone, the TMD alone, or both sites.

### Statistical analysis

Data analysis and visualization were performed in GraphPad Prism 8. Model figures (Figs. 1A; 4, A and B; 5A; 6, A and C; and 7, A to C) were made in Adobe Illustrator CS6. SMO structures (Figs. 1, B to G, and I; and 2, A, F, and G; and figs. S1C, S2A, S3A, and S3, C to E) were generated in PyMOL. Violin plots (figs. S1B and S2F) were generated with default settings in GraphPad Prism 8; outliers were excluded using the Identify Outlier function of GraphPad Prism 8 (ROUT method with a *Q* score = 10%). Mean and interquartile ranges for each plot are denoted by solid and dotted lines, respectively. Curve fitting (Figs. 2, D and E; and 5, C, E, and F; and fig. S6A) was carried out in GraphPad Prism 8 using a nonlinear regression fit and the log(agonist) versus response, variable slope (four parameters) option.

All statistical analyses comparing two datasets used a Student’s *t* test with Welch’s correction. When one dataset was compared to more than one dataset, an unpaired ordinary analysis of variance was used. All comparisons shown were prespecified. We note that a small sample size (*n* = 3) makes it difficult to assess whether the variance between different samples is comparable. Throughout the paper, the *P* values for the comparisons from GraphPad Prism 8 are denoted on the graphs according to the following key: not significant (ns) *P* > 0.05, *P ≤ 0.05, **P ≤ 0.01, ***P ≤ 0.001, and ****P ≤ 0.0001. Unless indicated otherwise, all experiments were performed three different times with similar results. Information about *n* values, replicates, experimental trials, and exact *P* values for each figure is provided below:

Replicates: In Figs. 2 (B and C), 3 (A to C and G), 4 (C, D, and F), 5 (B and D), and 6 (B, E, and F) and figs. S2 (G and H), S4 (A, C, and D), S5 (B and C), and S6E, bars denote the mean value derived from three independent measurements (biological replicates, *n* = 3). Each independent measurement is an average of two technical replicates. For all statistical tests, only the three biological replicates were considered (*n* = 3). In Figs. 2 (D and E) and 5 (C, E, and F) and fig. S6A, error bars denote the SEM from three independent biological replicates. In Fig. 2H and fig. S3B, shaded bars correspond to the SEM.

## Notes

View/request a protocol for this paper from *Bio-protocol*.
