# Supplementary material for: Patched 1 regulates Smoothened by controlling sterol binding to its extracellular cysteine-rich domain
Source: Sci Adv. 2022 Jun 3;8(22):eabm5563. doi: 10.1126/sciadv.abm5563 (PMC9166294; doi:10.1126/sciadv.abm5563)
Supplement: Supplementary file 1 — Figs. S1 to S6 Table S1 Supplementary Methods [file sciadv.abm5563_sm.pdf]

Science Advances
AAAS

Supplementary Materials for

Patched 1 regulates Smoothened by controlling sterol binding to its extracellular cysteine-rich domain

Maia Kinnebrew *et al.*

Corresponding author: Christian Siebold, christian.siebold@strubi.ox.ac.uk; Rajat Rohatgi, rrohatgi@stanford.edu

Sci. Adv. **8**, eabm5563 (2022)
DOI: 10.1126/sciadv.abm5563

This PDF file includes:

Figs. S1 to S6
Table S1
Supplementary Methods

Figure S1

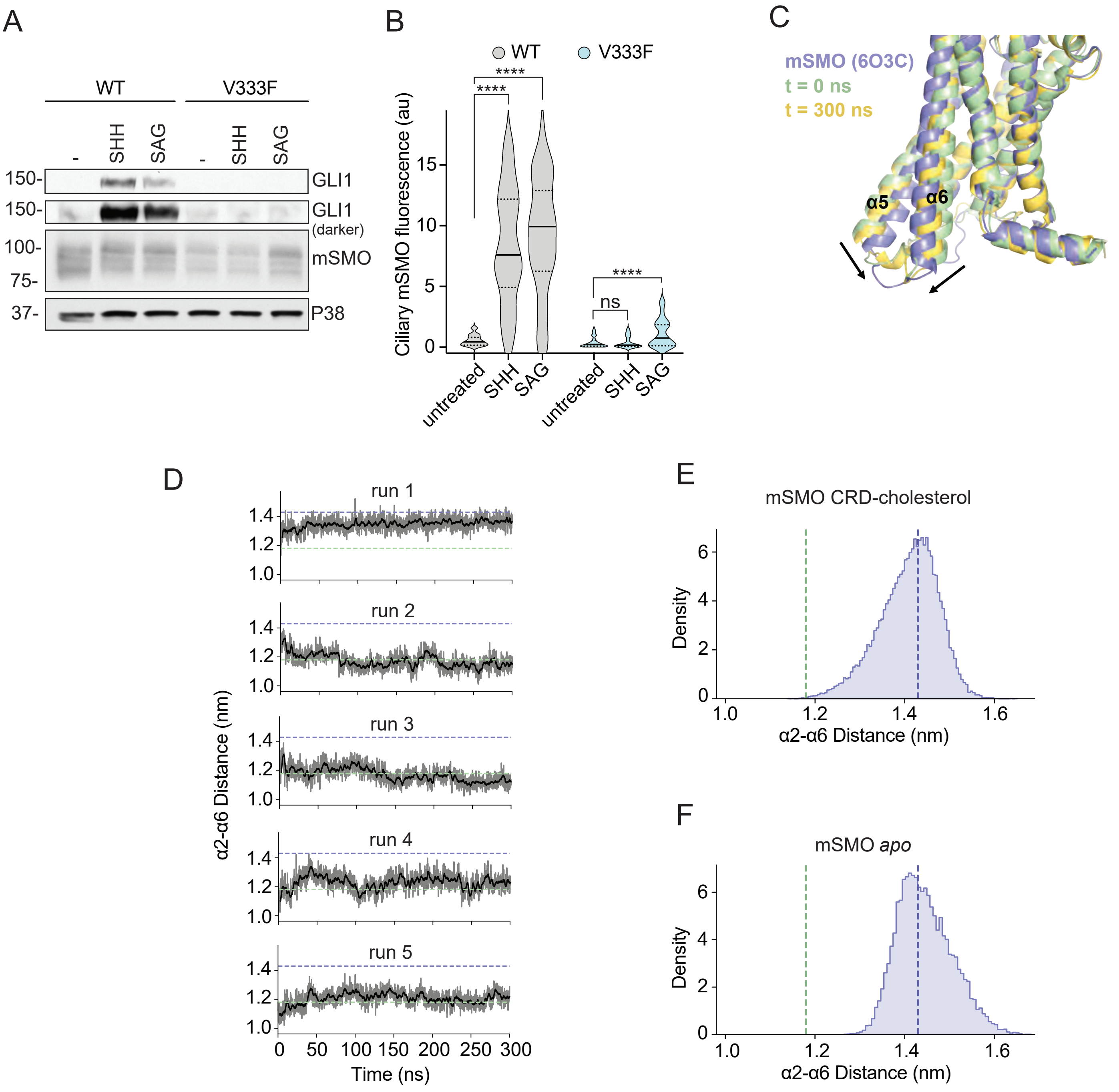
A
WT
V333F
-
SHH
SAG
150-
100-
75-
37-
GLI1
GLI1 (darker)
mSMO
P38
B
WT
V333F
Ciliary mSMO fluorescence (au)
****
ns
untreated
C
mSMO (6O3C)
t = 0 ns
t = 300 ns
α5
α6
D
run 1
run 2
run 3
run 4
run 5
α2-α6 Distance (nm)
Time (ns)
E
mSMO CRD-cholesterol
Density
α2-α6 Distance (nm)
F
mSMO apo

Figure S2

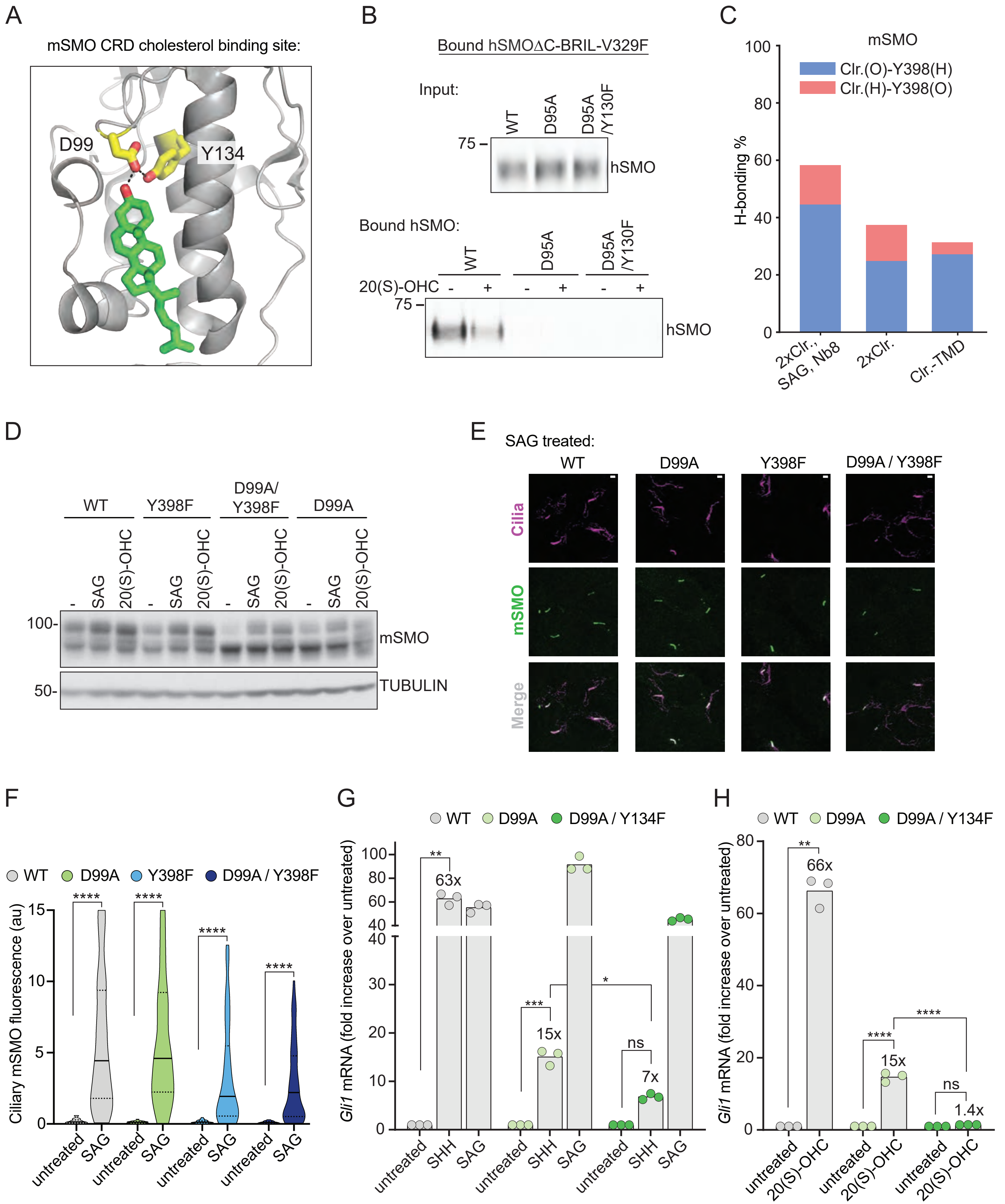

A
mSMO CRD cholesterol binding site:
D99
Y134
B
Bound hSMOΔC-BRIL-V329F
Input:
WT
D95A
D95A /Y130F
75
hSMO
Bound hSMO:
20(S)-OHC
75
hSMO
C
mSMO
Clr.(O)-Y398(H)
Clr.(H)-Y398(O)
H-bonding %
2xClr., SAG, Nb8
2xClr.
Clr.-TMD
D
WT
Y398F
D99A/ Y398F
D99A
SAG
20(S)-OHC
100-
50-
mSMO
TUBULIN
E
SAG treated:
WT
D99A
Y398F
D99A / Y398F
Cilia
mSMO
Merge
F
WT
D99A
Y398F
D99A / Y398F
Ciliary mSMO fluorescence (au)
untreated
SAG
G
WT
D99A
D99A / Y134F
Gli1 mRNA (fold increase over untreated)
63x
15x
7x
ns
untreated
SHH
SAG
H
WT
D99A
D99A / Y134F
Gli1 mRNA (fold increase over untreated)
66x
15x
1.4x
ns
untreated
20(S)-OHC

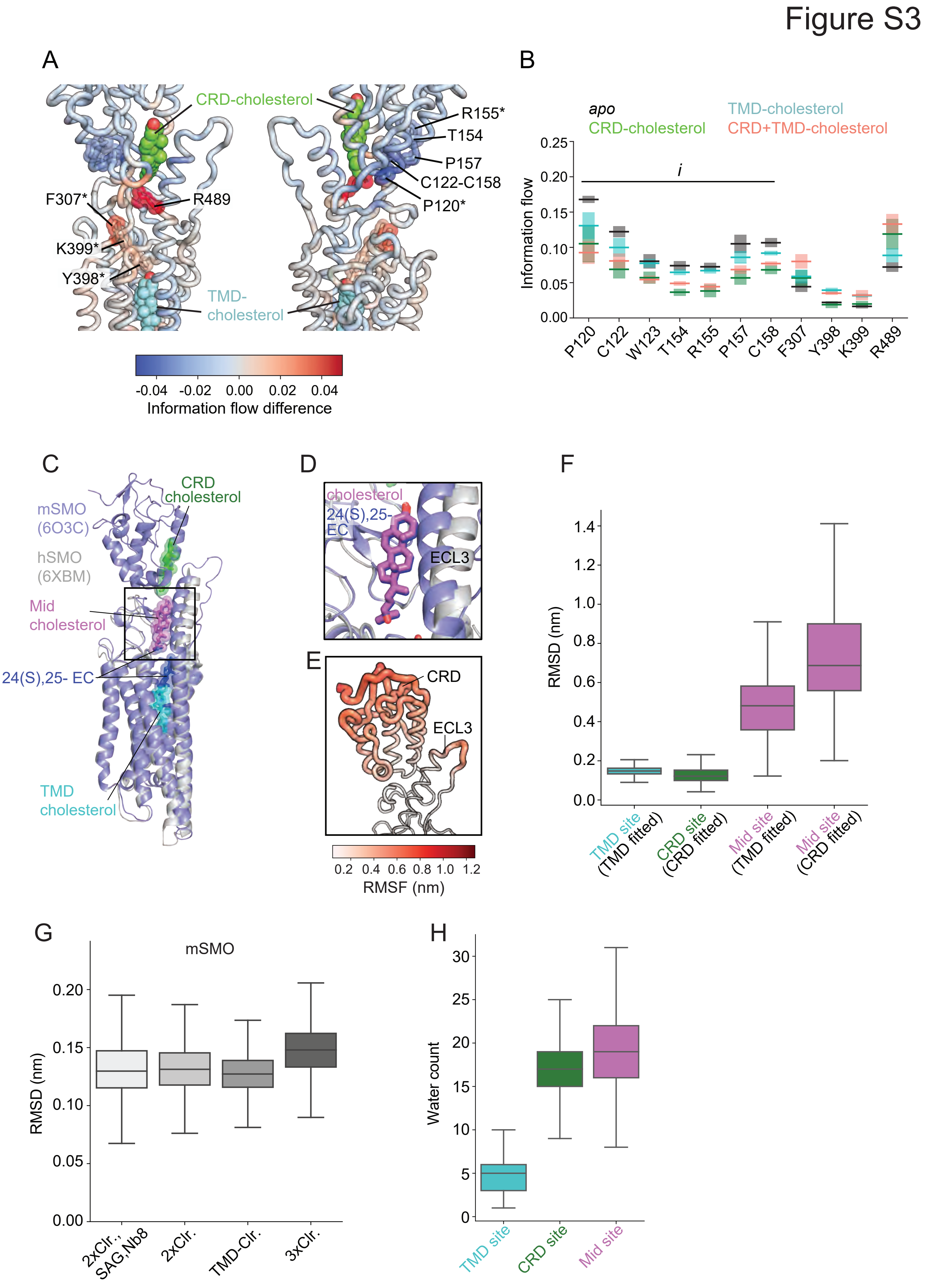
Figure S3
A
CRD-cholesterol
R155*
T154
P157
C122-C158
P120*
F307*
R489
K399*
Y398*
TMD-
cholesterol
-0.04 -0.02 0.00 0.02 0.04
Information flow difference
B
apo
CRD-cholesterol
TMD-cholesterol
CRD+TMD-cholesterol
Information flow
i
P120
C122
W123
T154
R155
P157
C158
F307
Y398
K399
R489
C
mSMO
(6O3C)
hSMO
(6XBM)
CRD
cholesterol
Mid
cholesterol
24(S),25- EC
TMD
cholesterol
D
cholesterol
24(S),25-
EC
ECL3
E
CRD
ECL3
0.2 0.4 0.6 0.8 1.0 1.2
RMSF (nm)
F
RMSD (nm)
TMD site (TMD fitted)
CRD site (CRD fitted)
Mid site (TMD fitted)
Mid site (CRD fitted)
G
mSMO
RMSD (nm)
2xClr., SAG,Nb8
2xClr.
TMD-Clr.
3xClr.
H
Water count
TMD site
CRD site
Mid site

Figure S4

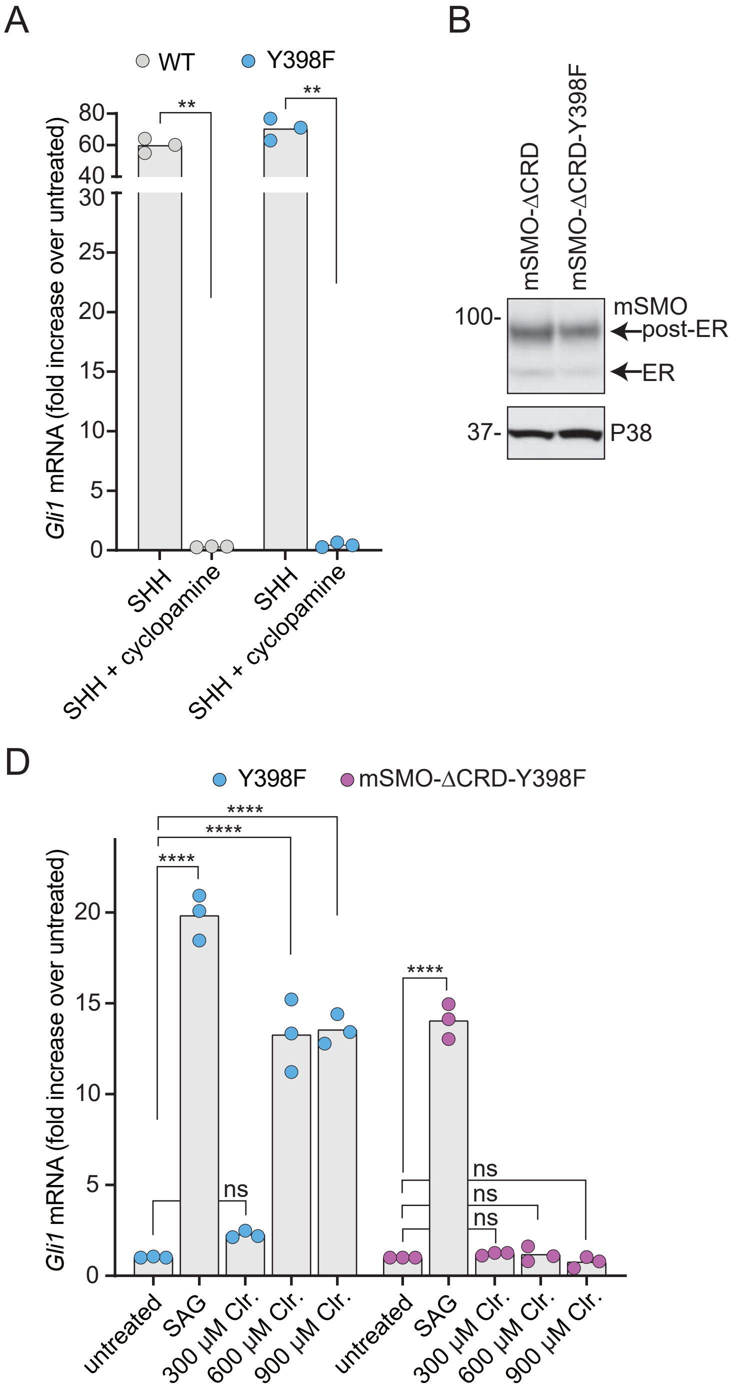
A
WT
Y398F
Gli1 mRNA (fold increase over untreated)
80
60
40
30
25
20
15
10
5
0
**
SHH
SHH + cyclopamine
B
mSMO-ΔCRD
mSMO-ΔCRD-Y398F
100-
mSMO
post-ER
ER
37-
P38
C
mSMO-ΔCRD
mSMO-ΔCRD-Y398F
***
0
0.25
2
SAG (μM)
D
Y398F
mSMO-ΔCRD-Y398F
****
ns
untreated
SAG
300 μM Clr.
600 μM Clr.
900 μM Clr.

Figure S5

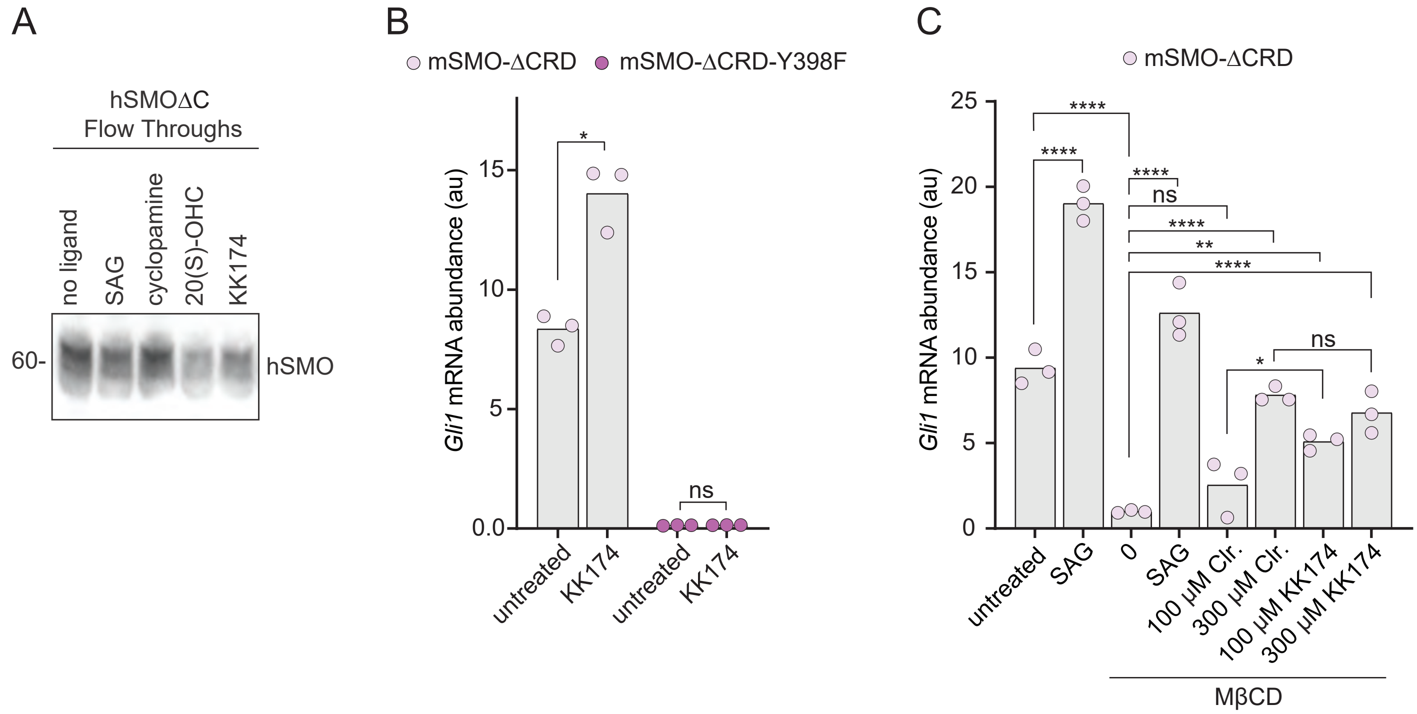

A
hSMOΔC
Flow Throughs
no ligand
SAG
cyclopamine
20(S)-OHC
KK174
60-
hSMO
B
mSMO-ΔCRD
mSMO-ΔCRD-Y398F
Gli1 mRNA abundance (au)
0.0
5
10
15
*
ns
untreated
KK174
untreated
KK174
C
mSMO-ΔCRD
Gli1 mRNA abundance (au)
0
5
10
15
20
25
****
****
****
ns
****
**
****
*
ns
untreated
SAG
0
SAG
100 µM Clr.
300 µM Clr.
100 µM KK174
300 µM KK174
MβCD

Figure S6

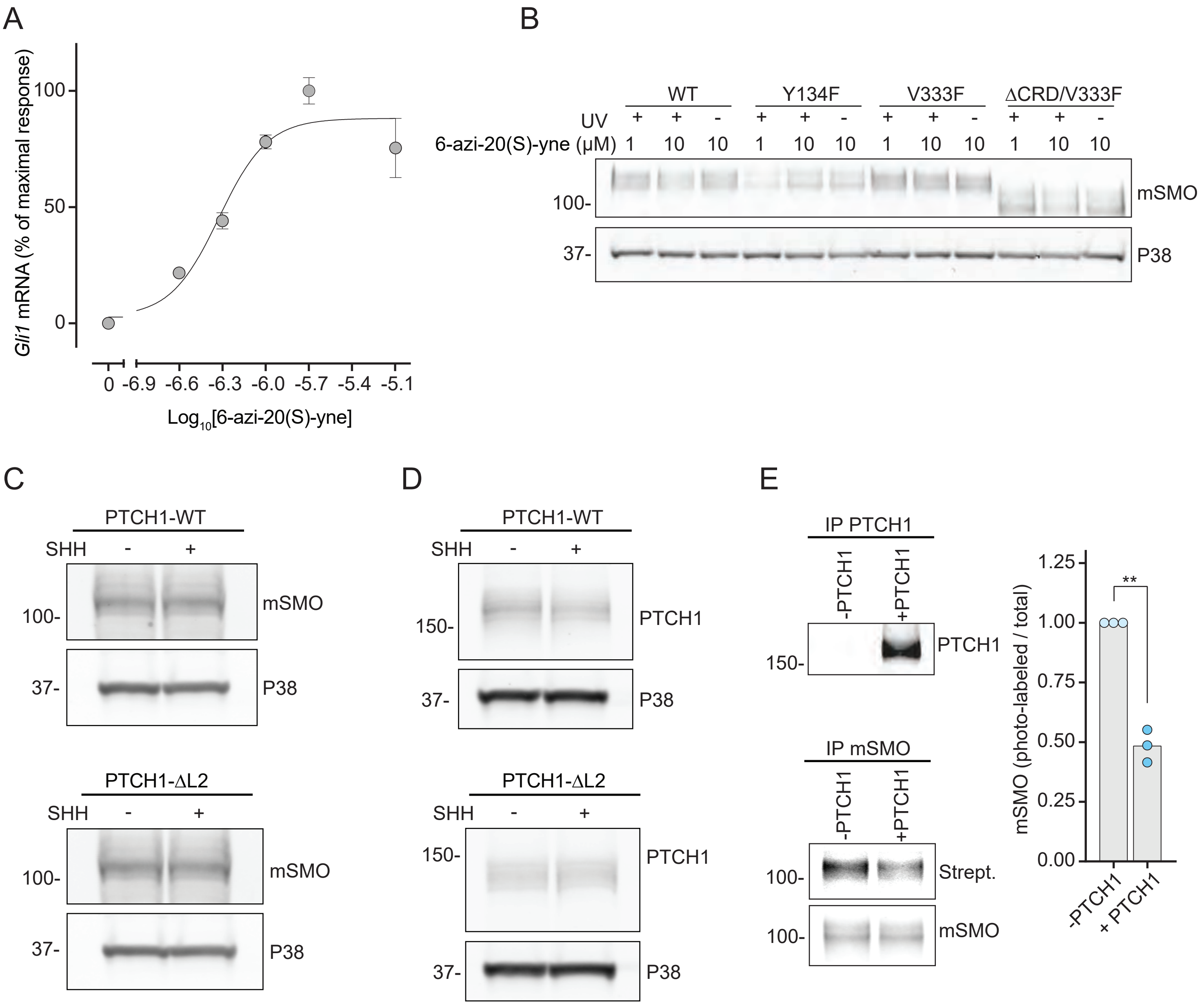
A
Gli1 mRNA (% of maximal response)
100
50
0
0 -6.9 -6.6 -6.3 -6.0 -5.7 -5.4 -5.1
Log10[6-azi-20(S)-yne]
B
WT
Y134F
V333F
ΔCRD/V333F
UV
6-azi-20(S)-yne (μM)
mSMO
P38
100-
37-
C
PTCH1-WT
SHH - +
mSMO
P38
100-
37-
PTCH1-ΔL2
D
PTCH1-WT
PTCH1-ΔL2
PTCH1
150-
E
IP PTCH1
-PTCH1
+PTCH1
IP mSMO
Strept.
mSMO (photo-labeled / total)
1.25
1.00
0.75
0.50
0.25
0.00
**
-PTCH1
+ PTCH1

Table S1. Data collection and refinement statistics for the SMO-SAG-cholesterol complex

**Data collection**	
Beamline	DIAMOND-I24
Wavelength (Å)	0.9686
No. crystals	3
Resolution (Å)	60.4-3.0 (3.08-3.00)
Space group	C2
Cell dimensions<br>a, b, c (Å)<br>α, β, γ (°)	 122.9, 63.2, 208.1<br>90.0, 96.3, 90.0
Unique reflections	31218 (2012)
Completeness (%)	97.1 (87.5)
R_{merge} (%)	9.7 (79.7)
R_{pim} (%)	8.2 (79.6)
$CC_{1/2}$	0.992 (0.363)
CC*	0.998 (0.730)
CC_{work}	0.925 (0.511)
CC_{free}	0.758 (0.612)
I/σI	9.9 (1.0)
Multiplicity	3.1 (2.3)
**Refinement**	
Resolution (Å)	60.0-3.0
No. reflections (test set)	29738 (1480)
R_{work}/R_{free} (%)	23.1/27.5
No. atoms<br>Protein<br>Ligand/Ion	 9246<br>163
B factors ($Å^2$)<br>Protein<br>Ligand/Ion	 134<br>112
r.m.s.d. bonds (Å)	0.014
r.m.s.d. angles (°)	1.641
Ramachandran (%)	
Favoured	96.3
Allowed	3.6
Disallowed	0.2
MolProbity score	1.59 (100th percentile; N=3130, 3.00±0.25 Å)
MolProbity clash score	6.21 (100th percentile; N=75, 3.00±0.25 Å)

Supplementary Methods

6-azi-20(S)-yne synthesis

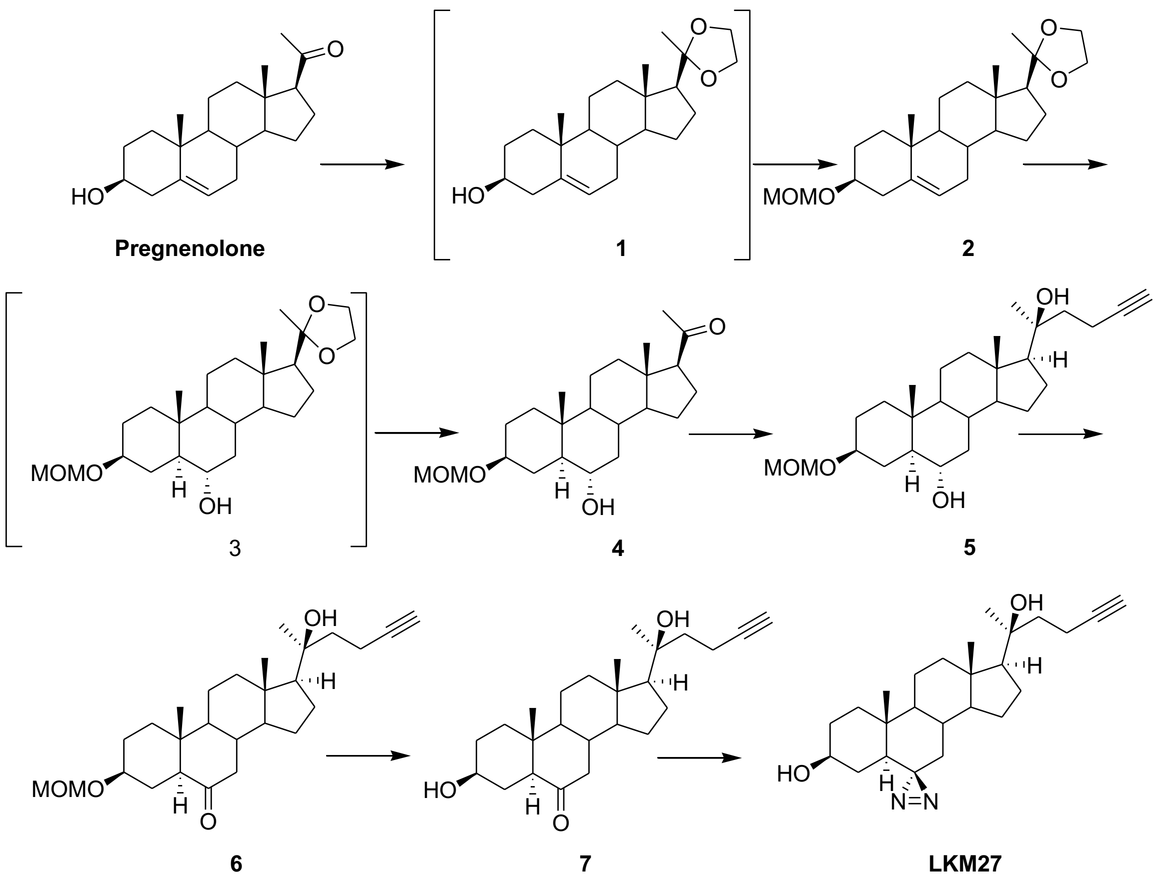

<u>(3β)-3-(Methoxymethoxy)-pregn-5-ene-20-one, cyclic 1,2-ethanediyl acetal (2):</u> Pregnenolone (2.0 g, 6.32 mmol) was dissolved in benzene (30 mL) in a round bottom flask fitted with a Dean Stark apparatus. Ethylene glycol (1.06 mL, 18.9 mmol) was added, followed by pyridinium *p*-toluenesulfonate (0.32 g, 1.26 mmol). The reaction was stirred at 110 ºC for 16 hours. Upon completion, the reaction mixture was diluted with dichloromethane (150 mL), and then washed sequentially with water, brine, dried over anhydrous Na_2SO_4 and solvents removed *in vacuo* for 2 hours. The crude residue **1**, was then redissolved in dichloromethane (75 mL) and cooled to 0 ºC. Diisopropylethylamine (4.4 mL, 25.3 mmol) was added, followed by chloromethyl methyl ether (0.96 mL, 12.6 mmol). After 10 minutes, the reaction mixture was warmed to room temperature and stirred for 16 hours. Upon completion, the reaction mixture was washed sequentially with water, brine, water, dried over anhydrous Na_2SO_4, and solvents removed *in vacuo*. The residue was purified by flash column chromatography (silica gel eluted with EtOAc–hexanes, gradient elution), to yield compound **2** in 94% yield (2.4 g, 5.93 mmol). Steroid **2** had: ^{1}H NMR (400 MHz, $CDCl_3$) δ 5.34 (d, 1H, *J* = 5.6 Hz, H-6), 4.67 (s, 2H,

CH_3OCH_2O), 4.05-3.81 (m, 4H, OCH_2CH_2O), 3.35-3.46 (m, 1H, H-3), 3.35 (s, 3H, CH_3OCH_2O), 1.28 (s, 3H, H-21), 1.00 (s, 1H, H-19), 0.77 (s, 1H, H-18); ^{13}C NMR (100 MHz, $CDCl_3$) δ 140.9, 121.8, 112.1, 94.9, 77.1, 65.3, 63.4, 58.4, 56.8, 55.3, 50.3, 42.0, 39.7, 39.6, 37.4, 36.9, 32.0, 31.6, 29.1, 24.7, 24.0, 23.1, 21.0, 19.5, 13.0.

(3β,5α,6α)-6-hydroxy-3-(methoxymethoxy)-pregnan-20-one (4): Steroid **2** (2.3 g, 5.7 mmol) was dissolved in THF (110 mL) at 0° C. 1 M BH_3 in THF (14 mL, 14.0 mmol) was added dropwise, and the temperature was allowed to rise to 20 °C over a period of 4 hours. Upon completion, the reaction mixture was cooled to 0 °C and a mixture of 10%NaOH (123 mL) and 30%H_2O_2 (165 mL) was added dropwise. The reaction was slowly warmed to 20 °C over 4 hours. The reaction mixture was then extracted with Et_2O (4 x 75 mL), dried over Na_2SO_4, and solvents removed *in vacuo*. Crude steroid **3** was then dissolved in acetone (30 mL). Pyridinium *p*-toluenesulfonate (0.20 g, 0.78 mmol) was added and the reaction mixture was refluxed at 56 °C for 16 hours. Upon completion, the acetone was evaporated, and the crude residue was dissolved in dichloromethane and washed sequentially with water, brine, water. The organic layer was then dried over Na_2SO_4, and solvents removed *in vacuo*. The residue was purified by flash column chromatography (silica gel eluted with EtOAc–hexanes, gradient elution), to yield steroid **4** in 33% yield (0.70 g, 1.85 mmol). Steroid **4** had: ^1H NMR (400 MHz, $CDCl_3$) δ 4.71-4.67 (overlapping doublets, 2H, *J* = 6.7 Hz, *J* = 6.7 Hz, CH_3OCH_2O), 3.55-3.37 (m, 2H, H-3, H-6), 3.37 (s, 3H, CH_3OCH_2O), 2.53 (dd, *J* = 9.7 Hz, *J* = 8.6 Hz, H-17), 2.12 (s, 3H, C(O)CH_3), 0.82 (s, 3H, H-19), 0.61 (s, 3H, H-18); ^{13}C NMR (100 MHz, $CDCl_3$) δ 209.7, 94.7, 76.1, 69.5, 63.9, 56.5, 55.4, 53.9, 51.9, 44.4, 41.7, 39.0, 37.5, 36.6, 34.4, 31.7, 29.4, 28.6, 24.6, 23.0, 21.3, 13.6, 13.6.

(3β,5α,6α,20*S*)-3-(Methoxymethoxy)-26,27-dinorcholest-24-yne-6,20-diol (5): Mg turnings (0.26 g, 10.6 mmol) were added to anhydrous Et_2O (20 mL). 1-bromo-4-trimethylsilyl-3-butyne (2.17 g, 10.6 mmol) was added followed by a few drops of 1,2-dibromoethane. The reaction mixture was warmed to 30 °C for 5 minutes with vigorous stirring, after which the reaction was allowed to cool back to room temperature and stirring was continued for another 30 minutes. The reaction was then cooled to 0 °C and steroid **4** (0.40 g, 1.06 mmol) dissolved in anhydrous THF (13 mL) was added dropwise. After 30 minutes at 0 °C, the reaction was brought to room temperature for an additional 10 minutes, but the reaction was found to proceed no further. Et_2O (25

mL) was added, followed by satd. aqueous NH_4Cl (~5 mL, or until the solution turned clear). The reaction mixture was then extracted with Et_2O (4 x 25 mL), the organic fractions were combined, dried over Na_2SO_4 and solvents removed *in vacuo*. The residue was purified by flash column chromatography on silica gel (eluted with EtOAc–hexanes, gradient elution), to yield the TMS-protected alkyne intermediate in 14% (76 mg, 0.15 mmol) yield, as well as 40% (160 mg, 0.42 mmol) recovered starting material. The alkyne intermediate was then immediately dissolved into THF (7 mL), and 1M TBAF in THF (0.33 mL, 0.33 mmol) was added dropwise. After 15 minutes, water (20 mL) was added, and the reaction mixture was extracted with EtOAc (3 x 10 mL). The organic extracts were combined, dried over anhydrous Na_2SO_4, solvents removed *in vacuo*, and the residue was purified by flash column chromatography (silica gel eluted with EtOAc–hexanes, gradient elution), to yield steroid **5** as a mixture of separable diastereomers (20*R*:20*S*) in 93% yield (60 mg, 0.14 mmol; 13% over two steps). Steroid **(20*R*)-5** was isolated in 10% yield (6 mg, 0.014 mmol) and steroid **(20*S*)-5** was isolated as colorless crystals in 83% yield (54 mg, 0.124 mmol). Steroid **(20*S*)-5** had: ^{1}H NMR (400 MHz, $CDCl_3$) δ 4.71-4.67 (overlapping doublets, 2H, *J* = 11.7 Hz, *J* = 11.7 Hz, CH_3OCH_2O), 3.55-3.40 (m, 2H, H-3, H-6), 3.37 (s, 3H, CH_3OCH_2O), 1.96 (t, 1H, *J* = 2.7 Hz, CCH), 1.27 (s, 1H, H-21), 0.83 (s, 3H) 0.82 (s, 3H); ^{13}C NMR (100 MHz, $CDCl_3$) δ 94.7, 85.1, 76.2, 74.9, 69.7, 68.6, 58.9, 56.5, 55.4, 53.9, 51.9, 43.2, 41.8, 41.7, 40.4, 37.5, 36.6, 33.8, 29.4, 28.6, 25.8, 23.9, 22.6, 21.2, 13.9, 13.6, 13.5.

<u>(3β,5α,20*S*)-20-Hydroxy-3-(methoxymethoxy)-26,27-dinorcholest-24-yne-6-one (6):</u> Steroid **5** (61 mg, 0.14 mmol) was dissolved in acetone (15 mL). Jones Reagent (~0.25 mL; 30% CrO_3·30% H_2SO_4·40% H_2O) was added dropwise until the solution remained a light brown color. At this time, a few drops of isopropyl alcohol were added to quench the remaining Jones Reagent, and the reaction solution was filtered through a short silica gel column, and eluted several times with EtOAc to remove retained chromium byproducts. The eluent was collected, solvents removed *in vacuo* and purified by flash column chromatography (silica gel eluted with EtOAc–hexanes, gradient elution), to give steroid **6** in 99% yield (60 mg, 0.14 mmol). Steroid **6** had: ^{1}H NMR (400 MHz, $CDCl_3$) δ 4.66 (overlapping doublets, 2H, *J* = 16.4 Hz, *J* = 16.4 Hz, CH_3OCH_2O), 3.39-3.52 (m, 1H, H-3), 3.35 (s, 3H, CH_3OCH_2O), 1.27 (s, 3H, H-21), 0.83 (s, 3H), 0.75 (s, 3H); ^{13}C NMR (100 MHz, $CDCl_3$) δ

210.8, 94.6, 85.0, 75.4, 74.8, 68.7, 58.9, 57.0 (2 x C), 55.4, 54.1, 46.7, 43.6, 41.7, 41.2, 40.1, 37.4, 36.9, 28.3, 27.1, 25.7, 23.7, 22.5, 21.5, 13.8, 13.4, 13.3.

(3β,5α,20*S*)-3,20-Dihydroxy-26,27-dinorcholest-24-yne-6-one (7): Steroid **6** (160 mg, 0.14 mmol) was dissolved into MeOH (5 mL), and 6 N HCl (2.5 mL) was added dropwise. The reaction was stirred for 8 hours at room temperature. The reaction was then neutralized with satd. aqueous $NaHCO_3$ and water (15 mL) was added. The reaction mixture was then extracted with dichloromethane (3 x 15 mL), and the organic fractions were combined, dried over anhydrous Na_2SO_4 and solvents removed *in vacuo*. The residue was purified by flash column chromatography (silica gel eluted with EtOAc–hexanes, gradient elution) to yield steroid **7** in 63% yield (90 mg, 0.23 mmol). Steroid **7** had: ^{1}H NMR (400 MHz, $CDCl_3$) δ 3.62-3.51 (m, 1H, H-3), 1.28 (s, 3H, H-21), 0.84 (s, 3H), 0.75 (s, 3H); ^{13}C NMR (100 MHz, $CDCl_3$) δ 211.0, 85.0, 74.8, 70.8, 68.7, 58.8, 57.0, 57.0, 54.0, 46.7, 43.6, 41.7, 41.1, 40.1, 37.4, 36.8, 30.8, 30.2, 25.7, 23.7, 22.5, 21.5, 13.8, 13.4, 13.3.

(3β,5α,20*S*)-Spiro-[26,27-dinorcholest-6,3'-[3*H*]-diazirin-24-yne]-3,20-diol (LKM27): Compound **7** (55mg, 0.14 mmol) was dissolved in MeOH:dichloromethane (3 mL:1 mL), and the reaction was cooled to 0 ºC. Ammonia gas was bubbled into the solution for 2 hours. Keeping the reaction at 0 ºC, the bubbling was stopped, additional dichloromethane (1 mL) was added, and the reaction was stirred for 2 hours. Hydroxylamine-*O*-sulfonic acid (80 mg, 0.71 mmol) dissolved MeOH (0.4 mL) was added dropwise. The reaction was stirred at 0 ºC for 10 minutes, brought to room temperature and stirred for an additional 16 hours. Upon completion, the reaction was poured into cold H_2O (10 mL), and extracted with dichloromethane (4 x 10 mL). The combined extracts were dried over anhydrous Na_2SO_4 and solvents were removed *in vacuo* to give a diaziridine intermediate. As the final diazarine compound is photo-reactive, the remainder of the synthesis was carried out under dimmed lights. The diaziridine was dissolved in dichloromethane (10 mL) and Et_3N (0.5 mL, 3.6 mmol) was added. MeOH saturated with molecular I_2 (5 mL) was added dropwise until a yellow color persisted. The reaction was immediately quenched with 10% aqueous $Na_2S_2O_3$ until colorless, and was then diluted with dichloromethane (10 mL), washed with water (2 x 5 mL), dried over anhydrous Na_2SO_4, and solvents removed *in vacuo*. The residue was purified by flash column chromatography (silica gel eluted with EtOAc–hexanes,

gradient elution), to yield **LKM27** as colorless crystals in 37% yield (21 mg, 0.05 mmol). **LKM27** had: mp 141–144 °C (from dichloromethane/hexanes); $[\alpha]_D^{20}$ = -10.07 (*c* = 0.14, $CHCl_3$); IR: 3306, 2935, 2849, 1582, 1444, 1382, 1051 cm^{-1}; ^1H NMR (400 MHz, $CDCl_3$) δ 3.51-3.41 (m, 1H, H-3), 1.96 (t, 1H, *J* = 2.6 Hz, CCH), 1.28 (s, 3H, H-21), 1.14 (s, 3H, H-19), 0.72 (s, 3H, H-18), 0.85-0.72 (m, 2H), 0.50-0.39 (m, 2H); ^{13}C NMR (100 MHz, $CDCl_3$) δ 85.1, 74.8, 71.2, 68.6, 58.9, 56.3, 53.8, 45.5, 43.3, 41.7, 40.3, 37.7, 37.4, 36.5, 33.4, 33.3, 30.9, 29.5, 25.8, 23.6, 22.5, 21.3, 13.9, 13.5, 13.2.9.

Supplementary Figure Legends

Figure S1. Analysis of SMO activation mechanisms using molecular dynamics.

(**A**) Immunoblotting was used to measure abundances of mSMO and GLI1 proteins in *Smo*$^{-/-}$ cells stably expressing either mSMO-WT or mSMO-V333F after treatment with either SHH or SAG. (**B**) Violin plots depict quantification of the ciliary accumulation of mSMO-WT or mSMO-V333F after treatment with SHH (50 nM, 20 hours) or SAG (100 nM, 20 hours). The medians (solid line) and interquartile ranges (dotted lines) are depicted within each violin. Statistical significance was determined using an ordinary unpaired ANOVA. (**C**) Concerted outward movement of α5 and α6 during one atomistic simulation (run 1 in **D**) of hSMOΔC bound to SAG and cholesterol at the CRD. The position of helices at the start (green, t=0 ns) and end (yellow, t=300 ns) of the simulation are shown, overlaid with an active-state structure of mSMO (PDB 6O3C, blue) (*13*). Another view of this helix movement is shown in **Figure 1I**. (**D**) Distances between the α2 and α6 helices across all five replicates of the hSMOΔC simulations. Dashed lines indicate α2-α6 distances at the start of hSMOΔC (green) and mSMO (blue) simulations. (**E** and **F**) Histogram of α2-α6 distances in atomistic simulations (5x 300 ns) of mSMO bound to CRD-cholesterol (**E**) or in a ligand-free *apo* state (**F**). Exact *p*-values for comparisons in **B**: mSMO-WT untreated vs. SHH< 0.0001, mSMO-WT untreated vs. SAG < 0.0001, mSMO-V333F untreated vs. SHH > 0.9999, mSMO-V333F untreated vs. SAG < 0.0001.

Figure S2. Analysis of SMO mutants bearing mutations in the CRD and the TMD.

(**A**) Close-up view of the cholesterol binding site in the mSMO CRD (PDB 6O3C) showing the hydrogen bonding network between the 3β-hydroxyl of cholesterol (green) and the D99 and Y134 residues of mSMO (yellow). (**B**) A ligand-affinity assay was used to measure the amount of wild-type, D95A (human numbering) or D95A/Y130F hSMOΔC-BRIL-V329F (*7*) captured on cholesterol-coupled beads (*14*). Binding reactions were performed in the presence or absence of a soluble CRD-binding competitor (50 μM 20(S)-OHC) to establish specificity. (**C**) Atomistic simulations (see methods) showing the frequency of hydrogen bonding between Y398 and the 3β-hydroxyl of the cholesterol molecule bound in the TMD site of mSMO. The 3β-hydroxyl of cholesterol can serve as the hydrogen bond acceptor (slate blue color) or donor (brick color). Simulations were performed in the presence of bound CRD-cholesterol, SAG and Nb8 (left bar), CRD-cholesterol (middle bar) or no additional ligands (right bar). All three simulations included the TMD cholesterol. (**D**) Immunoblotting was used to measure abundances of the indicated mSMO variants stably expressed in *Smo*$^{-/-}$ cells in the presence of indicated agonists (100 nM SAG, 5 μM 20(S)-OHC, treatment time 20 hours). (**E** and **F**) Immunofluorescence micrographs (**E**) showing the accumulation of mSMO-WT, mSMO-D99A, mSMO-Y398F or mSMO-D99A/Y398F in cilia after treatment with 100 nM SAG for 20 hours. Scale bar is 2 μm. Violin plots (**F**) depict quantification of ciliary accumulation. The medians (solid line) and interquartile ranges (dotted lines) are depicted within each violin. (**G** and **H**) SHH (50 nM), SAG (100 nM), or 20(S)-OHC (5 μM) triggered fold-increases in *Gli1* mRNA in *Smo*$^{-/-}$ cells stably expressing mSMO-WT, mSMO-D99A, or mSMO-D99A/Y134F treated with agonists for 20 hours. Exact *p*-values for comparisons: (**F**) are < 0.0001 for all mSMO variants. (**G**) mSMO-WT untreated vs. SHH = 0.0021, mSMO-D99A untreated vs. SHH = 0.0001, mSMO-D99A/Y134F untreated vs. SHH = 0.0839, mSMO-D99A vs. mSMO-D99A/Y134F (+SHH) = 0.0112. (**H**) mSMO-WT untreated vs. 20(S)-OHC = 0.0014, mSMO-D99A untreated vs. 20(S)-OHC < 0.0001, mSMO-D99A/Y134F untreated vs. 20(S)-OHC = 0.8332, mSMO-D99A vs. mSMO-D99A/Y134F (+20(S)-OHC) < 0.0001.

Figure S3. Analysis of allosteric pathways between the TMD and CRD sites by molecular dynamics. (**A**) Difference in information flow through mSMO between simulations where both the CRD- and TMD-sites were occupied by cholesterol compared to those where both sites were unoccupied (the ligand-free *apo* state). Blue indicates regions of reduced information flow compared to the *apo* state, as observed in the CRD and linker domains. Red indicates regions of increased information flow compared to the *apo* state, as seen in proximity to the TMD and ECL3. Residues shown to be important for SMO signaling by mutational analysis are starred. (**B**) Information flow through residues highlighted in (**A**) in simulations of mSMO initiated from an *apo* state (black), with a single cholesterol bound to the CRD (green) or TMD (cyan), or with both cholesterol sites occupied (red). Residues marked with *i* correspond to those on the CRD/linker shown in (**A**) which exhibited reduced information flow when compared to the *apo* state. (**C**) Overlay of the structure of hSMO (PDB 6XBM, grey (*16*)) bound to two 24(S),25-epoxycholesterol (24(S),25-EC, dark blue) molecules with a model of mSMO (blue) bound to three cholesterol molecules positioned in the TMD (cyan), CRD (green) and an intermediate site (Mid site, purple), which corresponds to the position of the upper 24(S),25-EC molecule. Cholesterol and 24(S),25-EC molecules are shown as sticks and transparent spheres. (**D**) Close up of the overlay of the 24(S),25-EC (blue) and cholesterol (purple) molecules positioned at the Mid site. (**E**) Root mean square fluctuation (RMSF) of the mSMO CRD during 5x 300 ns simulations of mSMO bound to the three cholesterol molecules. RMSF values are coloured from regions of low (white) to high (red) fluctuation to indicate destabilization of the CRD by the Mid site cholesterol (compared to **Fig. 2F**). The trajectories were fitted to the TMD. (**F**) Root mean square deviation (RMSD) of the TMD (cyan), CRD (green) and Mid (purple) site cholesterol molecules during atomistic simulations indicating the large degree of instability of the Mid site cholesterol compared to the CRD and TMD sites. (**G**) RMSD of the TMD cholesterol across atomistic simulations (5 x 300 ns each) of mSMO in complex with i) Nb8, SAG and cholesterol molecules bound to the TMD and CRD sites, ii) cholesterol bound to TMD and CRD sites, iii) cholesterol bound to the TMD site only or iv) cholesterol bound to the TMD, CRD and Mid sites. Simulations show destabilization of the TMD-cholesterol when the Mid site is occupied by cholesterol. (**H**) Solvent exposure of the TMD, CRD and Mid site cholesterol molecules across atomistic simulations of mSMO bound to all three molecules. Solvent exposure was defined as the number of waters within 0.4 nm of the site cholesterol, as calculated using MDAnalysis (*69*).

Figure S4. Analysis of mSMO-Y398F and mSMO-∆CRD-Y398F protein integrity.

(**A**) Fold-increase in *Gli1* mRNA abundance after 20 hour treatment with SHH (50 nM) or SHH in the presence of cyclopamine (5 µM) in *Smo*$^{-/-}$ cells stably expressing mSMO-WT or mSMO-Y398F. (**B**) Immunoblot showing expression of mSMO-∆CRD and mSMO-∆CRD-Y398F in stable cell lines. The top arrow indicates the mature form of the protein that has passed through the Endoplasmic Reticulum (ER) protein quality-control checkpoint and acquired the final glycosylation marks in the Golgi; the bottom arrow indicates the pool of protein in the ER. (**C**) Fold-increase in *Gli1* mRNA abundance after treatment with indicated concentrations of SAG for 20 hours in *Smo*$^{-/-}$ cells stably expressing mSMO-∆CRD or mSMO-∆CRD-Y398F. (**D**) Fold-increase in *Gli1* mRNA abundance after 20 hour treatment with SAG (100 nM), or indicated concentrations of MβCD:cholesterol (Clr.) in *Smo*$^{-/-}$ cells stably expressing mSMO-Y398F or mSMO-∆CRD-Y398F. Exact *p*-values for comparisons: (**A**) WT SHH vs. SHH + cyclopamine = 0.0020, Y398F SHH vs. SHH + cyclopamine = 0.0033. (**C**) mSMO-∆CRD untreated vs. 0.25 µM SAG = 0.0003, mSMO-∆CRD-Y398F untreated vs. 0.25 µM SAG = 0.0011. (**D**) Y398F untreated vs. SAG < 0.0001, Y398F untreated vs. 300 µM MβCD:cholesterol = 0.5191, Y398F untreated vs. 600 µM MβCD:cholesterol < 0.0001, Y398F untreated vs. 900 µM MβCD:cholesterol < 0.0001, mSMO-∆CRD-Y398F untreated vs. SAG < 0.0001, mSMO-∆CRD-Y398F untreated vs. 300 µM MβCD:cholesterol > 0.9999, mSMO-∆CRD-Y398F untreated vs. 600 µM MβCD:cholesterol > 0.9999, mSMO-∆CRD-Y398F untreated vs 900 µM MβCD:cholesterol > 0.9999.

Figure S5. Analysis of KK174 as a TMD agonist.

(**A**) Immunoblot showing hSMOΔC abundance in flow through fractions from the pulldown shown in **Figure 4B**.

(**B**) *Gli1* mRNA abundances in the absence of SHH (a measure of basal SMO activity) in *Smo*$^{-/-}$ cells stably expressing mSMO-ΔCRD or mSMO-ΔCRD-Y398F exposed to MβCD:KK174 (300 μM, 20 hours). (**C**) *Gli1* mRNA abundances in the absence of SHH (a measure of basal SMO activity) in *Smo*$^{-/-}$ cells stably expressing mSMO-ΔCRD. Where indicated, MβCD was added at 5 mM for 15 minutes to rapidly deplete cholesterol from cells. Media was then exchanged to 0.5% lipoprotein-depleted serum media containing 1 μM U18666A and 1 μM Lovastatin with or without the addition of SAG (100 nM), MβCD:cholesterol (100 or 300 μM), or MβCD:KK174 (100 or 300 μM) for 20 hours before analysis. Exact *p*-values for comparisons: (**B**) mSMO-ΔCRD untreated vs. KK174 = 0.0101, mSMO-ΔCRD-Y398F untreated vs. KK174 = 0.2899. (**C**) untreated vs. SAG < 0.0001, untreated vs. MβCD < 0.0001, MβCD vs. MβCD + SAG < 0.0001, MβCD vs. MβCD + 100 μM MβCD:cholesterol = 0.5194, MβCD vs. MβCD + 300 μM MβCD:cholesterol < 0.0001, MβCD vs. MβCD + 100 μM MβCD:KK174 = 0.0019, MβCD vs. MβCD + 300 μM MβCD:KK174 < 0.0001, 100 μM MβCD:cholesterol vs. 100 μM MβCD:KK174 = 0.0209, 300 μM MβCD:cholesterol vs. 300 μM MβCD:KK174 = 0.4451.

Figure S6. Analysis of 6-azi-20(S)-yne as a crosslinkable CRD-specific agonist.

(**A**) Dose-response curve for a 20 hour treatment of 6-azi-20(S)-yne in *Smo*$^{-/-}$ cells stably expressing mSMO-WT. Error bars denote the standard error of the mean from four measurements (two biological replicates with two technical replicates each). Based on the curve fit, the EC_{50} of 6-azi-20(S)-yne is 0.5 μM. (**B**,**C**,**D**) Immunoblots showing the abundances of mSMO or PTCH1 in total extracts (input samples prior to immunoprecipitation) for the experiments shown in **Figures 6D**, **6E** and **6F**, respectively. (**E**) Photo-crosslinking of mSMO to 6-azi-20(S)-yne in the presence or absence of PTCH1-WT expression. Immunoblot (top) shows a pulldown of PTCH1 from the two cell lines used. The graph (right) depicts the ratio of photo-labeled to total mSMO eluted from beads in three technical replicates. Statistical significance was determined by a Student's t-test. Exact *p*-value = 0.0058.
